# Lymphocyte antigen 6G6D-mediated modulation through p38α MAPK and DNA methylation in colorectal cancer

**DOI:** 10.1186/s12935-022-02672-1

**Published:** 2022-08-11

**Authors:** Francesca Pia Caruso, Mario Rosario D’Andrea, Luigi Coppola, Matteo Landriscina, Valentina Condelli, Luigi Cerulo, Guido Giordano, Almudena Porras, Massimo Pancione

**Affiliations:** 1 Bioinformatics Laboratory, BIOGEM scrl, Ariano Irpino, Avellino Italy; 2 UOSD Oncologia, Ospedale S. Paolo, 00053 Civitavecchia, Italy; 3 UOC Anatomia ed Istologia Patologica e Citologia Diagnostica, Dipartimento dei Servizi Diagnostici e della Farmaceutica, Ospedale Sandro Pertini, ASL Roma 2, 00157 Rome, Italy; 4 Laboratory of Pre-Clinical and Translational Research, IRCCS, Referral Cancer Center of Basilicata (CROB), Rionero in Vulture, Potenza Italy; 5 Unit of Medical Oncology and Biomolecular Therapy, Department of Medical and Surgical Sciences, University of Foggia, Policlinico Riuniti, 71122 Foggia, Italy; 6 Department of Biochemistry and Molecular Biology, Faculty of Pharmacy, Complutense University Madrid, 28040 Madrid, Spain; 7 Health Research Institute of the Hospital Clínico San Carlos (IdISSC), 28040 Madrid, Spain; 8 Department of Sciences and Technologies, University of Sannio, Benevento, Italy

**Keywords:** Lymphocyte antigen 6G6D, DNA methylation, Colorectal cancer, Anti-tumor immunity

## Abstract

In addition to being novel biomarkers for poor cancer prognosis, members of Lymphocyte antigen-6 (Ly6) gene family also play a crucial role in avoiding immune responses to tumors. However, it has not been possible to identify the underlying mechanism of how Ly6 gene regulation operates in human cancers. Transcriptome, epigenome and proteomic data from independent cancer databases were analyzed in silico and validated independently in 334 colorectal cancer tissues (CRC). RNA mediated gene silencing of regulatory genes, and treatment with MEK and p38 MAPK inhibitors were also tested in vitro. We report here that the Lymphocyte antigen 6G6D is universally downregulated in mucinous CRC, while its activation progresses through the classical adenoma-carcinoma sequence. The DNA methylation changes in *LY6G6D* promoter are intimately related to its transcript regulation, epigenomic and histological subtypes. Depletion of DNA methyltransferase 1 (DNMT1), which maintains DNA methylation, results in the derepression of *LY6G6D* expression. RNA-mediated gene silencing of p38α MAPK or its selective chemical inhibition, however, reduces LY6G6D expression, reducing trametinib’s anti-inflammatory effects. Patients treated with FOLFOX-based first-line therapy experienced decreased survival due to hypermethylation of the *LY6G6D* promoter and decreased p38α MAPK signaling. We found that cancer-specific immunodominant epitopes are controlled by p38α MAPKs signaling and suppressed by DNA methylation in histological variants with Mucinous differentiation. This work provides a promising prospective for clinical application in diagnosis and personalized therapeutic strategies of colorectal cancer.

**Supplementary Information:**

The online version contains supplementary material available at 10.1186/s12935-022-02672-1.

## Background

Colorectal cancer (CRC) is the third most commonly diagnosed cancer worldwide [1]. Although inherited genetic susceptibility plays a key role in a subset of cancers, the vast majority of CRCs are sporadic and not inherited [1, 2]. A Western diet, smoking, obesity, diabetes, alcohol consumption, and exposure to carcinogens are important risk factors for the development of sporadic CRC [2]. Early detection of CRC is critical for effective treatment. In recent years, genomic and high-throughput profiling of mRNA expression data from patient tissues have greatly improved our understanding of tumor biology and environmental factors leading to colon carcinogenesis [3]. The international CRC subtyping consortium has defined four molecular subtypes of CRC: consensus molecular subtypes (CMS)1 to CMS4, each of which has a distinctive molecular background with a different gene expression profile, mutation status and inflammatory microenvironment [3]. However, this stratification is not unambiguous, as even defined subtypes often exhibit intratumoral heterogeneity, with multiple subpopulations within a tumor showing differences in inflammatory infiltrates, mutations, and morphology [3, 4]. These studies have shown that CRC is not a uniform disease, but rather a heterogeneous collection of neoplastic diseases. A crucial contribution to the pathogenesis of CRC also depends on the anatomical location of the tumor and differs between right- and left-sided CRC [5, 6]. In routine clinical practice, CRC tissues can be distinguished by a large number of histopathological variants, but no reliable tissue-specific antigens are available to unambiguously identify these different variants [7]. In this context, we have recently identified lymphocyte antigen 6, member of the G6D family (*LY6G6D),* as a tissue-specific antigen that is unique to colorectal cancer and has minimal or relatively low expression in normal colorectal mucosa [8]. We know that *LY6G6D* is induced de novo in CRC, but the mechanism of its diffuse activation remains unclear. The LY6G gene cluster on human chromosomes 6 belongs to the major histocompatibility complex (MHC) class III. Recently, several genes encoding the III class region have been described and appear to be involved in both global and specific inflammatory responses [9]. It has been suggested that genes of the LY-6 family can be regulated in original ways, for example, by forming chimeric transcripts with their neighbors or undergoing an intron retention event, a mechanism that would prevent expression in most cell lines and tissues [10]. Thus, it might be possible that blockade of *LY6G6D* expression may be triggered only at a specific developmental stage or in specific pathological processes, but no conclusive data are available. In this study, we show that the *LY6G6D* gene is characterized by relatively high basal DNA methylation in both the promoter and gene body regions, and its expression is activated by DNA hypomethylation through the classical adenoma-carcinoma sequence. Conversely, *LY6G6D* expression is downregulated and its promoter hyper-methylated in mucinous CRC, regardless of anatomical location. In vitro studies and bioinformatic predictions revealed that p38α MAPK and DNA methyltransferase 1 (DNMT1) play important roles in controlling LY6G6D expression. Finally, we show that hypermetylation of the *LY6G6D* promoter may be a predictor of resistance to FOLFOX therapy, which is used as a basic scaffold for the treatment of patients with metastatic CRC.

## Methods

### In silico analysis of non-pathological and colorectal cancer datasets

Gene expression profiles, RNA-sequencing and clinical data were downloaded using the TCGA biolinks R/Bioconductor package [11]. To better correlate the expression of *LY6G6D* with distinct histological types, we analyzed an impendent dataset (affymetrix transcriptomic profiles, GSE4045) corresponding to serrated primary CRCs, which are morphologically different from conventional adenocarcinomas [12]. In total, 519 transcriptomic profiles of COAD, including 478 tumor and 41 normal samples, and 176 transcriptomic profiles of READ, including 166 tumor and 10 normal samples were collected from TCGA. To adjust gene-level effects and distributional difference within and between samples we applied the GC content correction and upper-quantile normalization to raw data using the EDASeq R/Bioconductor package [13]. All the differential expression analyses between two considered conditions were performed using the edgeR R/Bioconductor package [14]. Genes with an adjusted P-value (Benjamini & Hochberg correction) ≤ 0.01 and log2 Fold Change ≥|1.5| were considered significantly differentially expressed. To test if Gene Ontology-Biological Process terms show statistically significant differences between two biological states (i.e. tumor and normal) the CRC expression profiles were analyzed by GSEA, using the Cluster Profiler R/Bioconductor package [14]. Enrichment map was used for visualization of the GSEA results. Further, Gene Ontology-Biological Process terms overrepresentation analysis of the commonly up-regulated genes in tumor versus normal samples, both in COAD and READ, was computed using the Cluster Profiler R/Bioconductor package [15]. The Genotype-Tissue Expression (GTEx) biobank https://www.gtexportal.org/home/ was used to analyze normalized RNA-seq data indicated as Transcripts Per Million (TPM) from 54 nondisease tissue sites [16]. We collected data from human proteome atlas platform to integrate Transcriptomic data and the relative protein expression of the candidate genes. The subcellular and tissue distributions of proteins encoded by genes of interest were visualized by immunohistochemical (IHC) staining. High resolution IHC images and protein distribution across different cancer tissues were downloaded from https://www.proteinatlas.org/ [17]. The graphical representation of gene expression data and the derived survival curves were imported from GEPIA, a bioinformatics web tool for analyzing RNA sequencing expression data across TCGA [18]. Another interactive web resource named UALCAN, for analyzing cancer OMICS including Proteomic data from Clinical Proteomic Tumor Analysis Consortium (CPTAC), was used to identify or validate the correlation between gene expression data and pathological parameters [19]. The cBioPortal database (https://www.cbioportal.org/) was used to integrate mRNA expression, genomic data, somatic cell mutation, DNA methylation and DNA copy number changes in cancer samples [20]. The significance of genes in determining the overall survival was analyzed using the Kaplan–Meier curve. We considered as statistically relevant a p-value less than 0.05. To further establish the role of pathways involved in drug resistance, we analyze gene expression profiles and proteome data from oxaliplatin treated CRC patients and in vitro cellular models treated with and without oxaliplatin (GSE83129 and GSE83131) [21].

### Differential DNA methylation analysis

Comparison of the averaged methylation values was made between clinical groups at the CpG site level using Wilcoxon’s test for paired samples. DNA methylation analysis was performed using The Cancer Genome Atlas (TCGA) data and SMART App (http://www.bioin fo-zs.com/smart app), a web application for comprehensively analyzing DNA methylation data across TCGA project [22]. UALCAN mentioned above, was used as an independent platform to validate gene methylation in relation to cancer subtypes. To discriminate DNA methylation profiling the following criteria were used: β-difference > 0.2 and a FDR-corrected *p* value < 0.05. The Beta value indicates levels of DNA methylation ranging from 0 (unmethylated) to 1 (fully methylated). We considered Hyper-methylation and hypo-methylation levels based on that detected in normal non-neoplastic colonic mucosa. These same criteria were used to calculate the methylation difference among the CpG site level variants identified. The average of the β-values of differential CpG sites in the encoding regions and transcription start site (TSS) were used to establish the relationship between gene transcription and methylation profile. To ensure consistency of data processing, we compared samples with publically accessible samples with raw idat files. The human *LY6G6D* gene promoter and encoding regions were retrieved from http://genome.ucsc.edu/cgi-bin/hgGateway a genome, a genome browser to search and analyze genome sequence and annotation data. MethPrimer program at http://www.urogene.org was used to identify the distribution of CpG island in defined regions of LY6G6D structure. We also collected independent series of genome-wide DNA methylation datasets which included normal colonic mucosa, low-grade adenoma and high-grade adenoma (GSE139404) [23]. Genome-scale DNA methylation data (GSE148766) derived from in house primary-resistant or drug-sensitive mCRCs treated with 1st-line FOLFOX or FOLFIRI backbone chemotherapy were analyzed [24]. To establish whether the loss of three DNMTs, 1, 3A, and 3B, affect DNA methylation profiles and transcription at *LY6G6D* locus, we collected genome-wide DNA methylation and gene expression data from (GSE93136) [25]. Finally, 5-FU resistant cell line from its parental wild type CRC HCT-8 cell line treated both with 5-FU for 0 and 72 h and evaluated three replicates were assessed to verify the methylation pattern of LY6G6D using the Illumina 450 k Methylation Beadchip (GSE81006) [26].

### CRCs dataset of tissue microarrays analysis

Colorectal cancer tissues including a subset of adjacent normal tissues were obtained from the San Filippo Neri Hospital, Rome, Italy as previously reported [8]. The recruitment of samples was performed following the ethical guidelines, protocol number: 1703/2016 of September 2016 from the San Filippo Neri Hospital, Rome, Italy as already reported [8]. The procedure for tissue microarrays (TMAs) preparation and analysis was performed as previously described [8]. Briefly, the corresponding area on the matching formalin-fixed, paraffin-embedded tissue (donor block) was then identified and marked. Tissue cylinders with a 2 mm diameter were punched from representative tissue areas of each donor tissue block and placed into one recipient paraffin blocker. Each TMA spot included at least 50% tumor cells.

### Immunohistochemistry

Immunohistochemistry (IHC) analysis was performed as previously reported [8] using anti-LY6G6D (ab139649 Abcam, Cambridge, UK) on 4-μm-thick histological TMA sections. Mismatch repair (MMR) was investigated using the following antibodies: human mutS homolog 2 (anti-MSH2, ab92372), MutL homolog 1 (anti-MLH1, ab92312), human mutS homolog 6 (anti-MSH6, ab92471) from (Abcam Cambridge, UK). LY6G6D positive intrastromal cells were counted automatically with ImageJ-based software. All the cell counts were expressed as cells mm^−2^ as already reported [8]. The proportion of cancer cells stained was scored regardless of intensity as follows: Negative, as the complete absence of staining in more than 95% of tumor cells; Weak/moderate, characterized by a limited number of tumor cells scattered in a background of either negative or positive tumor cells; High or intense as a homogeneous staining in virtually all tumor cells.

### Cell lines, treatment and western blot analysis

The human colorectal carcinoma HCT116 cell line was obtained from ATCC (CCL-247) and authenticated by microsatellite markers analysis. According to reports [27], p38α shRNA was inserted into the pSuper.retro.puro vector to produce HCT116 cells that have p38α permanently knocked down. These cells were then selected with 2 g/ml puromycin. Cells transfected with the empty vector were also produced as a control. The SB203580 (Calbiochem; 559,389) was used to selectively inhibit p38α at concentration of 5 µM. The Trametinib, a selective MEK1/2 tyrosine kinase inhibitor, was used as described in [8]. The inhibitors were dissolved in sterile dimethylsulfoxide (DMSO) and a 10 mM working solution and stored in aliquots at − 20 °C. Working concentrations were diluted in culture medium just before each experiment. For western-blot analysis, cells were lysed in a buffer containing 50 mM Tris·HCl (pH 7.5), 150 mM NaCl, 1% NP40, 5 mM EGTA, 5 mM EDTA, 1 mM phenylmethylsulfonyl fluoride, 10 μg/ml aprotinin, 10 μg/ml leupeptin, 1 mM Na_3_VO_4_ and 20 mM NaF and centrifuged (at 13.000 rpm 10 min, 4 °C). Supernatants (total cell extracts) were stored at − 80 °C. Protein concentration was determined by the Bradford method. Western-blot analysis was carried out as previously described [8] using total cell extracts. Proteins were separated by electrophoresis using SDS-page gels and transferred to nitrocellulose membranes that were probed with the following antibodies against: P-p38MAPK (9211); P-ERKs (9101) Cell Signaling Technology and anti-LY6G6D (ab139649 Abcam, Cambridge, UK). β-actin, dilution 1:10,000, Sigma Aldrich) was used as loading control.

### Statistical analysis

The statistical analyses were carried out using Prism version 4.02 (GraphPad Software, Inc), GeneSpring R/bioconductor v.12.5 and R based package. All *p values* represent two-sided tests of statistical significance with p value < 0.05.

## Results

### *LY6G6D* is selectively and strongly expressed in colon and rectal adenocarcinomas

**Fig. 1:**
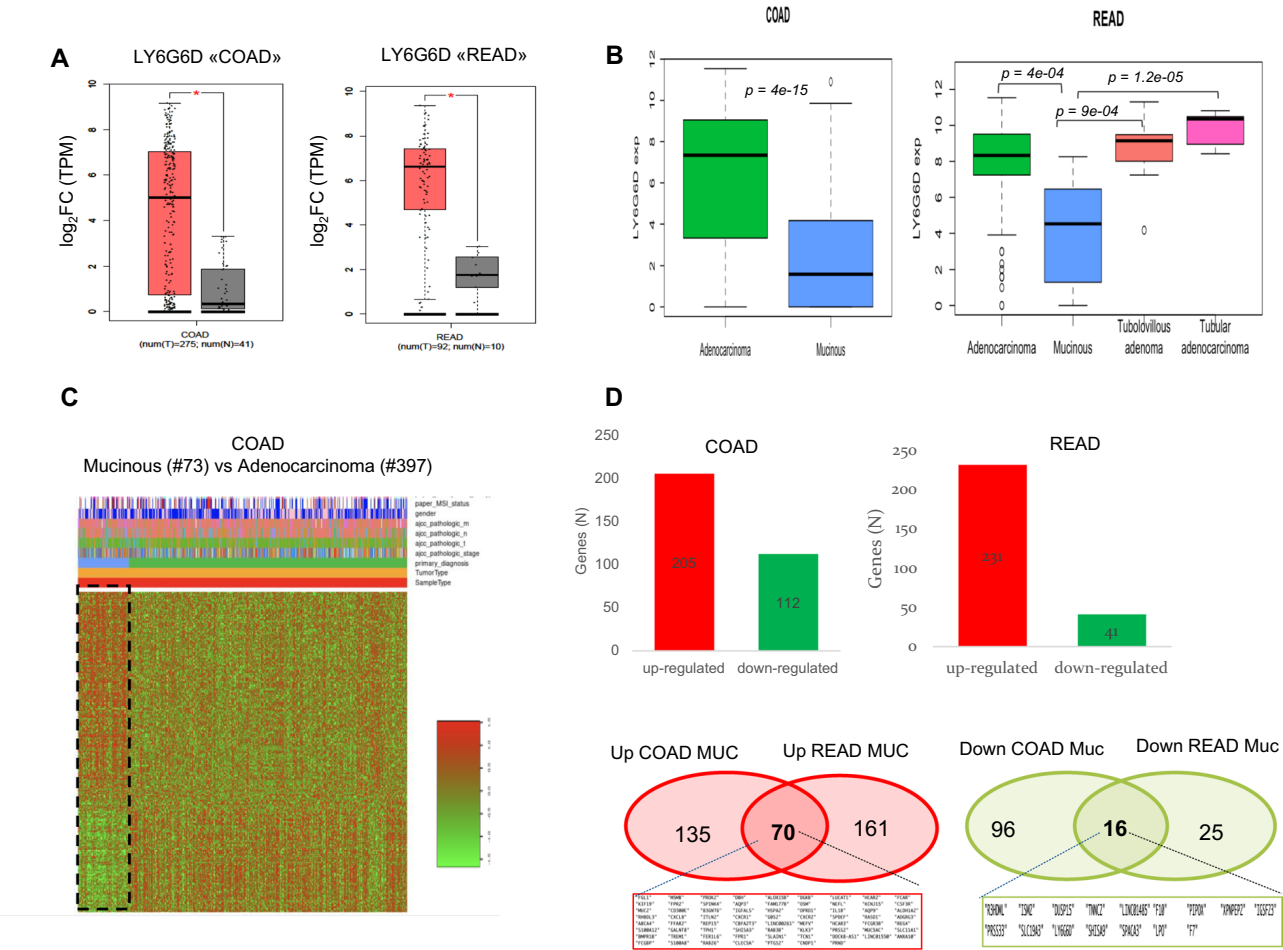
In silico TCGA analysis reveals differential *LY6G6D* expression in mucinous versus non-mucinous CRCs. **A** Quantification of *LY6G6D* mRNA in patient-matched tumor-normal mucosa extracted from The Cancer Genome Atlas (TCGA). **B** Left panel, the box plot shows *LY6G6D* mRNA expression levels in mucinous versus classical adenocarcinomas. Right panel, LY6G6D expression profile across classical adenocarcinomas and mucinous variants using for comparison Tubular Adenomas *p ≤ 0.05; t test Welch-corrected. **C** Heatmap of differentially expressed genes in mucinous versus Adenocarcinomas in CRC. **D** Upper panel, number of up and down-regulated genes in CRC according to the primary tumor location. Lower panel, shared up and down-regulated genes in both cancers. *COAD* colon adenocarcinoma, *READ* rectal adenocarcinoma

To comprehensively evaluate the gene expression profile of *LY6G6D*, we analyzed GTEx data with gene expression profiles from 54 nondiseased tissue sites. *LY6G6D* and its neighboring gene lymphocyte antigen 6 family member G6F (LY6G6F), as well as two other gut specific transcription factors encoding the Caudal Type Homeobox 1 and 2 genes, *CDX1* and *CDX2,* were included in the analysis [28]. As expected, *CDX1* and *CDX2* were mainly expressed in the small and large intestine. LY6G6D was most strongly expressed in testicular and prostate tissues, and moderate expression was found in the normal colon tissue “sigmoid colon”. In contrast, *LY6G6F* was most strongly expressed in blood, followed by testis, but not in colonic tissue (Additional file 1: Fig. S1A, B). These results suggest that *LY6G6D/6F* gene expression is restricted to specific human tissues, and that normal colon tissues are characterized by weak to moderate expression of *LY6G6D*. We next analyzed the expression profiles of *LY6G6D* and *LY6G6F* in tumor tissues from 31 different types in TCGA, using GTEx data for comparison. In tumor tissues, the highest expression of *LY6G6D* was in colon carcinoma, and *LY6G6D* was significantly overexpressed only in colon tumor tissues. The expression of *LY6G6D* was five times higher in colorectal carcinomas than in the corresponding normal mucosa (Fig. 1A and Additional file 1: Fig. S1C). Of note, the expression of *LY6G6F* was mainly found in CRC tissue, but on average, the expression in tumors was less than one-fold higher that that in normal tissue (Additional file 1: Fig. S1C). Averall, the in silico data showed consistent upregulation of *LY6G6D* found only at CRC.

### Downregulation *of LY6G6D* characterizes mucinous colon adenocarcinoma

**Fig. 2:**
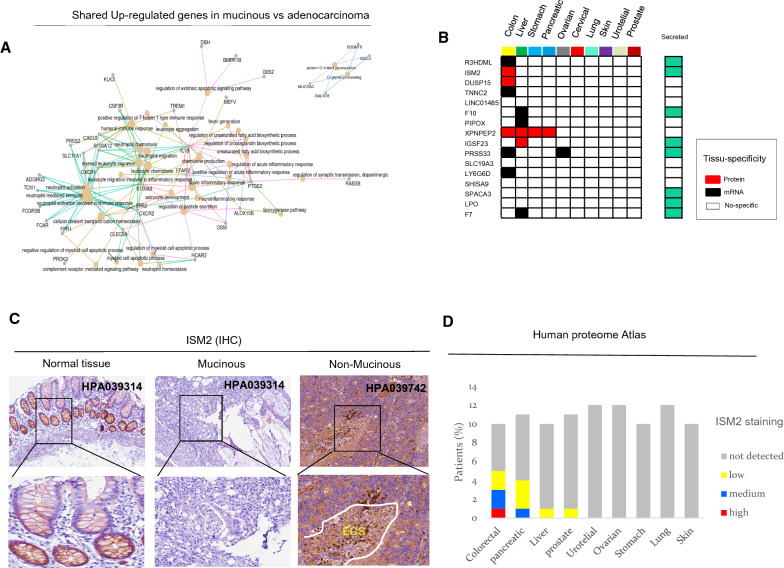
A down-regulated gene signature alters the secretome-based immune responses in mucinous CRC. **A** Over representation of the analysis of shared up-regulated genes in Mucinous COAD and READ. **B** bioinformatics prediction shows that shared down-regulated genes in Mucinous include a number of secreted proteins, often selectively up-regulated in CRC at protein (red square) and mRNA level (black square). **C** IHC images of ISM2 labeling in normal, mucinous and classical CRC adenocarcinoma. **D** IHC staining profiling of ISM2 (isthmin-like protein 1) across different cancers derived from human proteome atlas database. *ECS* extracellular space

To decipher the variations of LY6G6D expression in different histological subtypes of colorectal carcinoma, we examined the gene expression profiles in the TCGA database, which includes samples of colon adenocarcinoma (COAD, N = 478) and rectal adenocarcinoma (READ, N = 166), respectively. We found that the expression levels of LY6G6D were significantly lower in mucinous adenocarcinomas (MAD) than in classic colon adenocarcinomas (CAD), regardless of anatomic location (Fig. 1B). The low expression pattern of LY6G6D in mucinous cancer resembled that in normal colonic mucosa (Additional file 1: Fig. S1D). Interestingly, a clear overlap was observed between classical adenocarcinoma and tubular/villous adenoma, suggesting that upregulation of LY6G6D occurred early in adenoma and persisted after malignant transformation following the well-defined sequence from adenona to adenocarcinoma (Fig. 1B). By examining gene expression profiles in serrated colorectal carcinomas, we recapitulated the results obtained in TCGA and found that mucinous subtypes had lower expression of LY6G6D compared with non-mucinous colorectal carcinomas, even within specific histological variants (Additional file 1: Fig. S1D). Mucinous CRC is a specific histological subtype commonly associated with the proximal colon and characterized by dedifferentiation and mucin production [29]. Because LY6G6D has not previously been associated with differences in histotype, we analyzed the global gene expression profiles of mucinous and classical adenocarcinomas in the COAD and READ TCGA databases. Using a stringent FDR of 0.01, 205 up-regulated and 112 down-regulated genes were identified in COAD. On the other hand, 231 and 41 genes were up- and down-regulated in READ, respectively (Fig. 1C, D, Additional file 1: Fig. S2A and Additional file 2: Table S1, Additional file 3: Table S3). Overlapping the above results, a total of 70 genes were upregulated and 16 downregulated in mucinous CRC compared with classical adenocarcinoma. In particular, we found that the common downregulated cluster included the gene LY6G6D (Fig. 1D). In addition to the well-known MUC2 gene involved in mucin processing and secretion, analysis of the upregulated genes revealed pathways not previously known to be overexpressed in mucin cancer, including inflammatory chemokines such as CXC motif chemokine ligand (CXCL) 8, CXCR1 and CXCR2 receptor, and IL -1β-inflammosome [30] (Fig. 2A). Our analysis of the downregulated genes revealed biological changes in immunity (LY6G6D), protein digestion and absorption (XPNPEP2), lysine degradation (PIPOX), and vitamin digestion and absorption (SLC19A3) (Additional file 1: Fig. S2B). Mucinous dedifferentiation was associated with the CIMP-H/MSI phenotype, a high frequency of BRAF and KRAS mutations, and fewer changes in the p53 pathway, consistent with the literature [29] (Additional file 1: Fig. S2C, D, S3A). Thus, these data clearly indicate that LY6G6D is one of the genes that are generally downregulated in mucinous CRC.

### Proteogenomic profiling reveals DNA methylation changes in Mucinous gene signature

**Fig. 3:**
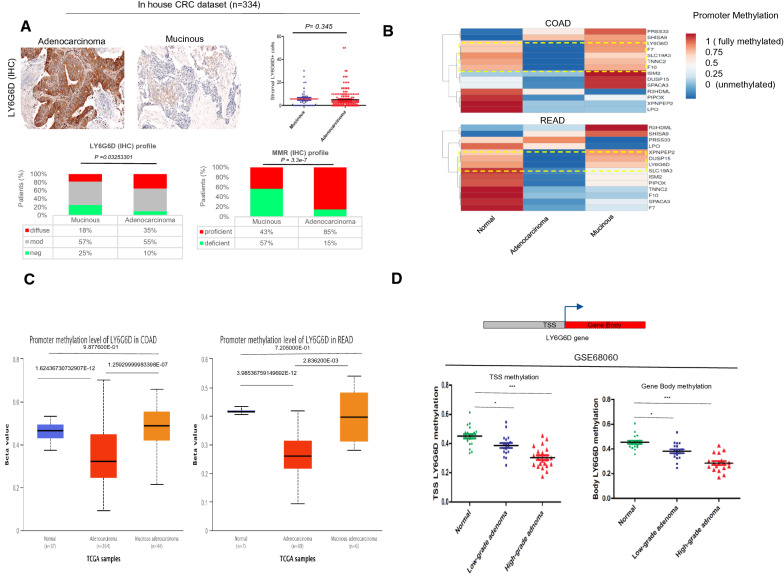
LY6G6D is epigenetically regulated in mucinous and classical adenocarcinoma. **A** Up panel, representative IHC pictures of LY6G6D staining in Mucinous and non-Mucinous CRC. Down, LY6G6D and MMR repair immunohistochemical profile in mucinous and classical adenocarcinoma in our CRC database. Up right, stromal quantification of LY6G6D + cells in mucinous versus non-mucinous CRC. **B** Heatmaps of differentially methylated genes related to Mucinous gene signature in COAD and READ dataset from TCGA. DNA methylation levels are expressed as beta value ranging from 0 (unmethylated, blue) to 1 (fully methylated, purple). **C** The boxplots show promoter methylation levels of LY6G6D in Mucinous and non-Mucinous CRC in COAD and READ dataset. p-values from t test Welch-corrected are shown. **D** DNA Methylation profile at the transcription start site (TSS) and gene body of the *LY6G6D* locus in normal colonic mucosa, low grade and high-grade adenomas, data extrapolated from GSE68060 database

To further explore the role of down-regulated genes, we integrated mRNA and protein expression data by immunohistochemistry (IHC) from the Human Protein Atlas. We found that approximately 50% of the genes encode secreted proteins (Fig. 2B). A subcluster of genes encoding proteases (PRSS33), protease inhibitors (R3HDML), and cytoskeletal factors (TNNC2) was overexpressed only in colon tumor tissues and resembled the expression pattern of LY6G6D (Additional file 1: Fig. S3B, C). For example, the IHC data showed intense extracellular positivity of ISM2 in classical adenocarcinomas compared with mucinous tumors, reflecting the differences in gene expression profiles (Fig. 2C, D). To validate the differential expression of LY6G6D in different CRC subtypes, we examined our in-house CRC tissue databases. Overall, we evaluated the immunohistochemical expression of LY6G6D in (n = 334) colorectal cancer tissues consisting of 51 (15%) mucinous and 283 (85%) non-mucinous adenocarcinomas, respectively. More than 50% of the malignant tissues showed moderate LY6G6D positivity regardless of histological type. However, negative staining was more common in mucinous carcinomas than in non-mucinous carcinomas (25% versus 10%). In contrast, we found that strong/intense membranous/cytoplasmic immunoreactivity of LY6G6D was less frequent in mucinous carcinomas than in non-mucinous carcinomas CRC, (18–35%) (p < 0.05) (Fig. 3A). All cases were also analyzed for mismatch repair protein expression by IHC. More than 50% of mucinous tumors were MMR-deficient, compared to 15% of classical adenocarcinomas, which is consistent with the literature [29]. Next, we examined the tumor microenvironment for infiltration of LY6G6D + cells in mucinous and non-mucinous tumors. We found no relevant difference in the intrastromal expression of LY6G6D (Fig. 3A). These results suggest that changes in LY6G6D expression are primarily related to cancer cell-intrinsic properties in CRC tissues. We next inquired about the causes of downregulated expression. Of note, in classical carcinomas, there was coamplification and overexpression of the R3HDML, TNNC2, and DUSP15 locus at chromosome band 20q11-13, suggesting functional cooperation between these coamplified genes (Additional file 1: Fig. S3D). In contrast, we found no relevant genetic abnormalities in mucinous CRCs. In light of these data, we examined the effects of DNA methylation variations in COAD and READ samples using TCGA data. We found that the majority of genes exhibited high basal DNA methylation in normal colonic tissue and similarly in mucinous CRC. In contrast, a striking 70% of genes in classic adenocarcinomas exhibited DNA hypomethylation. Remarkably, we found that only LY6G6D was methylated differently in mucinous tissue than in adenocarcinoma, regardless of the primary tumor site (Fig. 3B, C). Therefore, to obtain a more detailed picture, we examined the DNA methylation profiles of the entire LY6G6D gene. We examined three sequences: region (A) upstream of the transcription start site (TSS); region (B) overlapping with TSS; region (C) located in the coding region, the “gene body” (Additional file 1: Fig. S4A). We found consistent changes in DNA methylation patterns at the TSS and gene body levels (Additional file 1: Fig. S4B). Specifically, the methylation changes around TSS showed a strong and negative linear relationship with the transcription levels of the gene LY6G6D (R = − 0.91, p = 2.2e-16, Additional file 1: Fig. S4C).

To better understand whether epigenetic changes in LY6G6D play a role in CRC carcinogenesis, we analyzed DNA methylation changes at TSS and in the gene body in an independent data set that included normal colonic mucosa and low- and high-grade adenomas. We found that DNA methylation reduced considerably from normal mucosa to the transition from low-grade to high-grade adenomas, showing that LY6G6D hypomethylation is an early step in the progression of classical adenoma-carcinoma (Fig. 3D). This is confirmed by the fact that CIMP-negative CRCs (clusters 3 and 4) have higher expression of LY6G6D than CIMP-positive (CIMP-L and CIMP-H) tumors. A similar trend was also observed in a subset of 17 CRC cell lines (Additional file 1: Fig. S4D). These data suggest that changes in GpG island methylation have critical effects on LY6G6D transcriptional regulation and mark distinct CRC subtypes.

### The expression of *LY6G6D* is positively regulated by *p38α MAPK *(MAPK14) and repressed by DNA methyltransferase 1 (DNMT1)

**Fig. 4:**
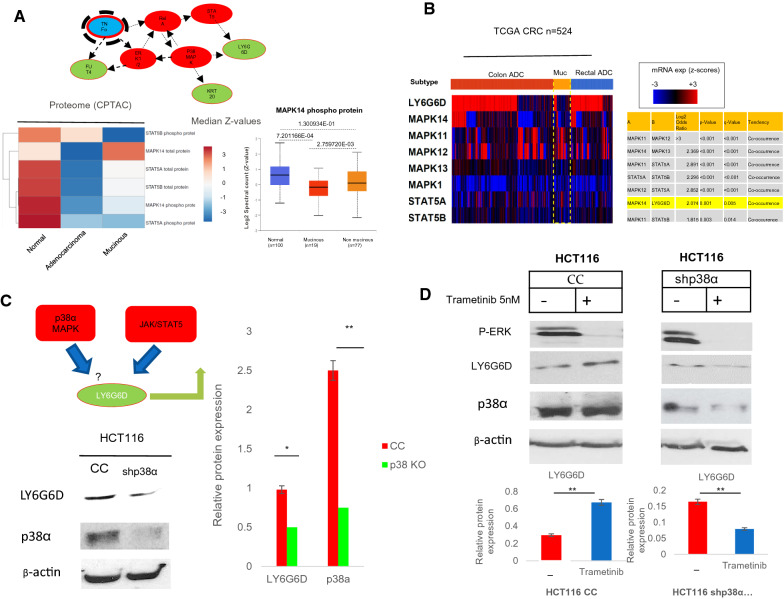
p38α MAPK mediated regulation of LY6G6D. **A** Upper panel, a network analysis reveals a putative role of TNF-α (blue) and p38α MAPK (red) in the regulation of LY6G6D. Down left, Heatmap of candidates LY6G6D regulatory proteins in mucinous and classical adenocarcinoma from the Clinical Proteomic Tumor Analysis Consortium (CPTAC). Down right, MAPK14 expression in normal, mucinous and non-mucinous CRC. The p values are from t test Welch-corrected. **B** Left panel, Heatmap of CRC transcriptomic changes for the candidate genes in mucinous and adenocarcinoma from TCGA dataset. Right, the table shows significant co-modification of p38α MAPK (MAPK14) and LY6G6D (yellow). **C** Left panel, western blot analysis of LY6G6D and p38α MAPK protein normalized to β-actin in control (CC) transfected with empty vector or p38α MAPK knock-down (shp38α) HCT116 cells. Right panels, densitometry analysis normalized to β-actin and, expressed as mean and standard deviation (SD) performed on triplicate experiments. *p < 0.05, **p < 0.01. **D** Upper panels, western blot analysis of P-ERK1/2, LY6G6D and p38α normalized to β-actin in extracts from the HCT116 control or p38α knock-down treated with the MEK inhibitor, trametinib, or untreated (−). Lower panel, histograms show the mean and SD performed on triplicate blots and normalized to β-actin for LY6G6D levels. *p < 0.05, **p < 0.01

**Fig. 5:**
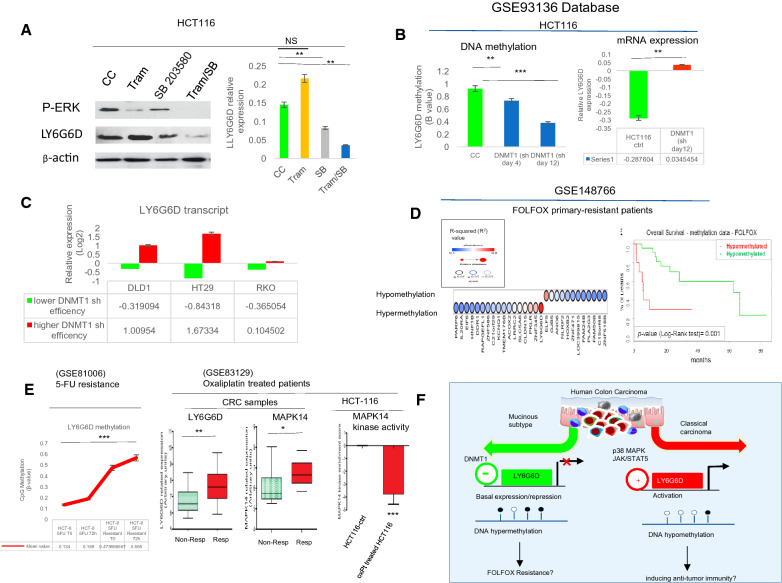
Reduced expression of LY6G6D and MAPK14 in response to FOLFOX therapy. **A** Left panel, western blot analysis of P-ERK1/2 and LY6G6D normalized to β-actin in cell extracts from HCT116 cells treated with SB203580 (5 nM) alone or in combination with trametinib. Right, histograms show the mean and SD of blots repeated three times and normalized to β-actin, *p < 0.05, **p < 0.01, ***p < 0.001. **B** Left, DNA methylation profile of *LY6G6D* promoter at days 4 and 12 following DNMT1 shRNA knockdown in HCT116 cells and control cells (CC); (β values between 0 and 1; y-axis). Right, expression profile of *LY6G6D* at day 12 (RNA-seq data) in cells subject to DNMT1 shRNA knockdown. p-values from t test, **p < 0.01, ***p < 0.001*.* **C** *LY6G6D* expression profile in the indicated CRC cell lines following DNMT1 knockdown with higher and lower shRNAs efficiencies. **D** Let, Dot plot for the top ranking hyper and hypo-methylated genes predicting resistance to FOLFOX therapy in our database GSE148766. Right, DNA methylation profile and overall survival analysis in Patients treated with FOLFOX therapeutic regime. **E** Left, CRC cell lines treated with 5-FU (GSE81006) were assessed to verify the methylation pattern at the *LY6G6D* locus. Histograms show the mean and SD of replicates experiments, ***p < 0.001. Right, The box plots show the expression profiles of LY6G6D and MAPK14 in primary tumour from 26 patients who were treated with oxPt and 5-FU as first-line therapy (GSE83129). The patients are classified as Responders (Resp) and non-Responders (non-Resp), respectively. Left, phosphoproteome data related to p38/MAPK14 phosphorylation extrapolated from HCT116.ctrl cells and HCT116-oxPt resistance (GSE83129). **F** The schematic depicts the pathways controlling LY6G6D expression, linked to tumor immunity and genotoxic cellular responses in CRC. Mucinous tumours are characterized by DNA hypermethylation and consequent reduction of *LY6G6D* expression. Conversely, DNA Hypomethylation and up-regulation of LY6G6D occurs in classic adenocarcinoma

We have previously shown that LY6G6D is positively regulated in a context-specific manner by the JAK /STAT5 pathway [8], but the molecular network involved in controlling its expression remains unknown. Using a regulatory network analysis strategy, we demonstrated a possible role of p38 MAPK in controlling LY6G6D expression (Fig. 4A). By integrating transcriptome and proteome data from TCGA, we found that the expression of p38α MAPK (MAPK14) tended to be lower in mucinous than in nonmucinous CRC at the protein and mRNA levels (Fig. 4A, B). Transcriptomic data showed a close association between MAPK14 (p38α MAPK) and LY6G6D gene expression in samples from CRC and derived cell lines, suggesting a possible co-regulation (Fig. 4B and Additional file 1: Fig. S5A). We therefore analyzed LY6G6D protein expression in HCT116 cells with permanent p38α knockdown (p38α shRNA), using cells transfected with the empty vector as a control [27]. LY6G6D expression decreased by approximately 50% in HCTC116 cells with p38α silencing compared to control cells (Fig. 4C). Since our previous results have shown that the STAT5/LY6G6D axis mediates resistance to the MEK inhibitor trametinib [8], we monitored LY6G6D expression levels after trametinib treatment in p38α-silenced HCT116 and control cells. As shown in (Fig. 4C, D) the level of the p38α protein was reduced by around 60–70% in p38α KD compared to control cells, and the levels of p38α were considerably lower after trametinib treatment. Remarkably, the data for LY6G6D levels showed similar trends. After trametinib treatment, we found increased LY6G6D levels in HCT116 control cells. Trametinib treatment on the other hand reduced the expression of LY6G6D in p38α knockdown cells (Fig. 4D). To further investigate the role of p38α in the regulation of LY6G6D, we treated HCT116 wild-type cells with SB203580, a known p38α inhibitor, in the presence or absence of trametinib. Treatment with SB203580 alone or in combination with trametinib resulted in downregulation of LY6G6D protein, suggestive of p38 MAP kinase-dependent regulation of LY6G6D expression levels (Fig. 5A). We next examined publicly available DNA methylation profiles and transcriptomic data in which the three DNMTs 1, 3A, and 3B were inactivated by the combination of genetic and shRNA silencing strategies. In HCT116 cells, we found that time-dependent shRNA-mediated silencing of DNMT1 reduced DNA methylation in the LY6G6D promoter four-fold compared with control. Loss of DNA methylation in DNMT1 knockdown cells was associated with significant reexpression of the LY6G6D transcript from − 0.28 to 0.034 compared with control (Fig. 5B). Analysis of additional colorectal cancer cell lines, DLD1, HT29, and RKO, after DNMT1 knockdown with higher and lower shRNA efficiencies confirmed DNMT1-dependent repression of the LY6G6D transcript (Fig. 5C). In contrast, genetic deletion of DNMT3A or 3B did not significantly affect LY6G6D promoter methylation, but double knockout (with DNMT1 and DNMT3B knockout) again drastically reduced DNA methylation (Additional file 1: Fig. S5B). Therefore, maintenance of DNA methylation by DNMT1 and p38α MAPK signaling play opposite roles in transcriptional regulation and control of LY6G6D expression.

### Epigenetic suppression of *LY6G6D* predicts poor outcomes in colon cancer chemotherapy

Mucinous colon cancer is associated with resistance to chemotherapy compared with nonmucinous colon cancer. Although the location of the primary tumor may play a role, the reason for this disparity is unclear [29]. A global analysis of TCGA clinical data revealed that CRC patients with low LY6G6D expression had shorter survival than those with high expression (Additional file 1: Fig. S5C). Surprisingly, we found that patients with low LY6G6D expression had shorter overall survival than patients with higher LY6G6D expression in rectal but not in colonic adenocarcinoma (Additional file 1: Fig. S6A). Our immunohistochemical analysis using the in-house database revealed that tumors with negative LY6G6D staining in left-sided tumors were associated with shorter survival than cases with moderate and high expression, but not in right-sided CRCs (Additional file 1: Fig. S6A). Collectively, these data suggest that epigenetic alterations at the LY6G6D locus may be associated with clinical and molecular features of CRC. Because LY6G6D has been associated with response to therapies, we analyzed our internal database of metastatic colorectal carcinomas that were primarily resistant to FOLFOX or FOLFIRI to understand whether the DNA methylation profile in LY6G6D might play a role in response to chemotherapy-based treatments. Surprisingly, the analysis revealed that hyper-methylation of LY6G6D was the most important predictor of resistance to FOLFOX-based therapy (Fig. 5D). Remarkably, LY6G6D hypermethylation was associated with a CIMP-high phenotype, demonstrating the association between DNA methylation changes and chemotherapy resistance (Additional file 1: Fig. S6C, D). Since 5-fluorouracil (5 FU) and oxaliplatin are the mainstay therapy of CRC, we analyzed high-throughput DNA methylation analysis in a cell line resistant to 5 FU and its parental wild-type cell line CRC HCT-8. We found that DNA methylation in *LY6G6D* promoter was significantly higher in cells 5-FU resistant (around three times higher) than in wild type (Fig. 5E). To better understand the potential mechanism by which oxaliplatin resistance is linked to changes in *LY6G6D*, high-throughput transcriptomic and proteomic data were obtained from Patients and CRC cell lines treated with oxaliplatin. We found that LY6G6D expression was downregulated in non-responders compared to those who responded to oxaliplatin treatment, and notably correlated with reduced expression or activity of MAPK14 (Fig. 5E). These data suggest that the *DNA* hyper*-*methylation at the LY6G6D gene and reduced activity of MAPK14 impair therapeutics to FOLFOX-based treatment.

## Discussion

Over the past years, advances in cancer immunology have radically changed therapeutic strategies for the treatment of many types of solid tumors, including colorectal cancer. So far, it is well documented that immune checkpoint inhibitors are effective in a small number of mismatch repair deficient (MMRd) malignancies [3, 4, 31]. Stable mismatch repair cancers account for the majority of CRCs and exhibit primary immune checkpoint resistance. Unfortunately, targeted therapies and immunotherapy have only shown their benefits in a limited subset of patients. Thus, it is necessary to define the molecular, immunological and biological landscape of each patient with CRC [31, 32]. LY6G constitute a gene cluster belonging to the MHC class III. The functions of many LY6G genes are still unknown, their molecular alterations in human diseases remain largely unexplored [9]. Recently, our group discovered LY6G6D as a tumor-specific antigen of stable mismatch repair CRCs [8]. Here, we show that expression of LY6G6D is activated following the classical adenoma-carcinoma sequence but downregulated in mucinous CRC, regardless of primary tumor site. Consistently, IHC profiling in our CRC database has clearly demonstrated that low expression of *LY6G6D* is typical of Mucinous CRC. In an attempt to explain the biological basis of our observations, we found that DNA methylation changes in the *LY6G6D* promoter are intimately linked to its transcriptional variations. It is well known that Mucinous tumors arise and progress through different molecular pathways [29]. They often exhibit mutations in *KRAS* and *BRAF*, microsatellite instability and a CpG island methylator phenotype. In addition to JAK/STAT5 signaling, our studies support a p38α-dependent regulation of LY6G6D, expanding the spectrum of inflammatory pathways that control its expression. In particular, we define a novel role for DNMT1 in maintaining DNA methylation and transcriptional repression of LY6G6D. In fact, downregulation of DNMT1 determines the loss of DNA methylation in *LY6G6D* promoter and its reexpression, in keeping with the model in (Fig. 5F). However, our findings raise an intriguing question about the impact of such changes in tumor-associated epigenetic mechanisms on malignant progression. Although the interaction between mucinous CRC and the host immune system is unknown, one might hypothesize that the pathways regulating LY6G6D may differentially compromise anti-tumor immunity at the site of the primary tumor [6]. DNA hypomethylation at the LY6G6D locus occurs in vast majority of CRCs and could be an “early warning system” to stimulate anti-tumor immune response, as for other immune antigens [33]. In the clinical setting, we found that *LY6G6D* hypermethylation predicted resistance to FOLFOX. Furthermore, acquired resistance to 5-FU induces hypermethylation of the LY6G6D promoter in CRC cells, confirming recent observations that DNMT inhibitors given in combination with standard with 5-FU or oxaliplatin improve therapeutic responses in patients with CRC [34]. Moreover, it has been reported that DNMT inhibitors can reshape the tumor microenvironment by increasing intratumoral T cells in vivo*,* and by prolonging survival of patients with stable mismatch repair CRCs [35]. Our data suggest that epigenetic control of LY6G6D may influence the response to chemotherapy via DNMT1 and p38α MAPKs signaling. Accordingly, a recent study showed that oxaliplatin antagonizes p38α MAPK signaling via miR-625-3p in CRC [21]. In conclusion, LY6G6D is a tissue-specific CRC antigen regulated by epigenetic mechanisms. DNMT1 and p38α MAPK signaling play important roles in controlling LY6G6D expression. These results could be relevant for the design of personalized therapeutic strategies in patients with CRC.

## Supplementary Information

**Additional file 1:** Supplementary figures 1-6.

**Additional file 2:** Table S1. Differentially expressed genes Mucinous vs Adenocarcinoma in COAD.

**Additional file 3:** Table S2. Differentially expressed genes Mucinous vs Adenocarcinoma in READ.

## Data Availability

Our in house DNA methylation data from (primary-resistant versus drug-sensitive tumors) have already been deposited at the NCBI GENE expression Omnibus repository (GEO) and are accessible through the accession number GSE148766. We collected independent genome-wide DNA methylation dataset from (GSE139404) and genome-wide DNA methylation and gene expression data from (GSE93136). Tissue microarrays, modified cell lines analysed during the current study are available from the corresponding author on reasonable request.

## Abbreviations

CRC: Colorectal cancer
LY6G6D: Lymphocyte antigen 6 complex, locus G6D
*BRAF*: B-rapidly accelerated fibrosarcoma
*ERK*: Extracellular-signal regulated kinase
KRAS: Kirsten rat sarcoma viral oncogene homolog
*MAPK*: Mitogen-activated protein kinase
*TCGA*: The cancer genome atlas
*IHC*: Immunohistochemistry
*GEO*: Gene expression omnibus
*GSEA*: Gene set enrichment analysis
CIN: Chromosomal instability
MMR: Mismatch repair
CIMP: CpG island methylator phenotype
JAK: Janus kinase
STAT: Signal transducer and activator of transcription
*ERK*: Extracellular-signal regulated kinase
MSI: Microsatellite instability
FOLFOX: Folinic acid (leucovorin, FOL), fluorouracil (5-FU, F), and oxaliplatin (Eloxatin, OX)
FOLFIRI: Leucovorin calcium (calcium folinate), 5-fluorouracil (5-FU), and irinotecan (IRI)
DNMT: DNA methyltransferase
MHC-III: Major histocompatibility complex class III
MEK: Mitogen extracellular kinase
