# Supplementary material for: Lymphocyte antigen 6G6D-mediated modulation through p38α MAPK and DNA methylation in colorectal cancer
Source: Cancer Cell Int. 2022 Aug 11;22:253. doi: 10.1186/s12935-022-02672-1 (PMC9373545; doi:10.1186/s12935-022-02672-1)
Supplement: Supplementary file 1 — Additional file 1: Supplementary figures 1-6. [file 12935_2022_2672_MOESM1_ESM.pdf]

# Figure S1

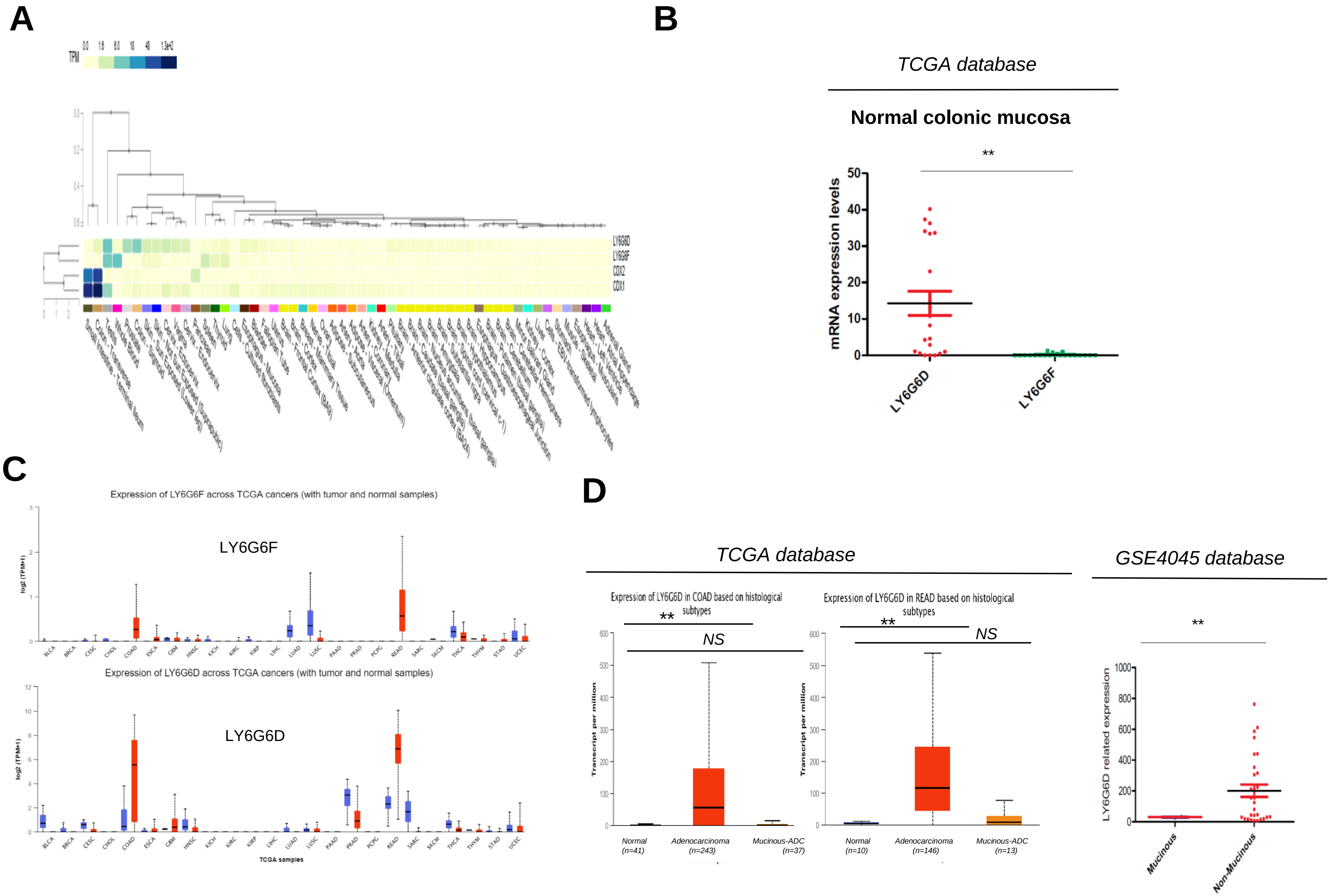


**Figure S1. LY6G6D/6F expression profiles in colorectal cancer. A)** differential mRNA expression profile of the indicated genes across 54 non-diseased Human tissues from Genotype-Tissue Expression (GTEx) platform. **B)** Differential expression of LY6G6F and LY6G6D mRNA in normal colonic tissues from TCGA***P≤0.05*; t test Welch-corrected. **C)** Differential *LY6G6F and LY6G6D* mRNA expression across TCGA cancer types (red) using for comparison matched non-tumor tissues (blue). **D)** Left panels, LY6G6D mRNA expression in TCGA across normal, adenocarcinoma and mucinous CRC in colon adenocarcinoma (COAD) and rectal adenocarcinoma (READ) using UALCAN platform.. Right, expression of *LY6G6D* n Mucinous vs non-Mucinous CRC in a database of serrated CRCs.. ***P≤0.01*; t test Welch-corrected

# Figure S2

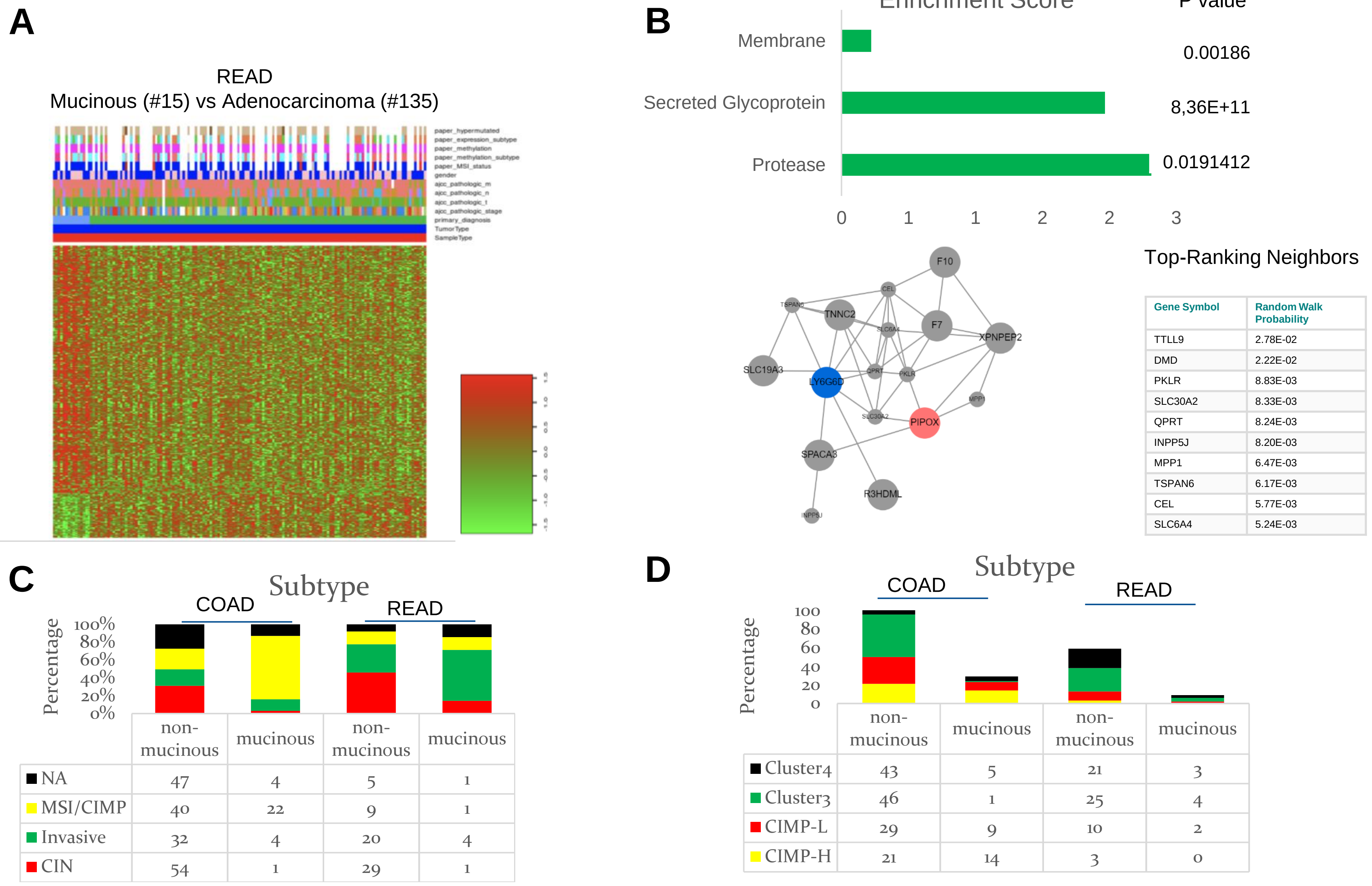


Top-Ranking Neighbors

| Gene Symbol | Random Walk Probability |
|---|---|
| TTLL9 | 2.78E-02 |
| DMD | 2.22E-02 |
| PKLR | 8.83E-03 |
| SLC30A2 | 8.33E-03 |
| QPRT | 8.24E-03 |
| INPP5J | 8.20E-03 |
| MPP1 | 6.47E-03 |
| TSPAN6 | 6.17E-03 |
| CEL | 5.77E-03 |
| SLC6A4 | 5.24E-03 |

C)

| | non-mucinous | mucinous | non-mucinous | mucinous |
|---|---|---|---|---|
| NA | 47 | 4 | 5 | 1 |
| MSI/CIMP | 40 | 22 | 9 | 1 |
| Invasive | 32 | 4 | 20 | 4 |
| CIN | 54 | 1 | 29 | 1 |

D)

| | non-mucinous | mucinous | non-mucinous | mucinous |
|---|---|---|---|---|
| Cluster4 | 43 | 5 | 21 | 3 |
| Cluster3 | 46 | 1 | 25 | 4 |
| CIMP-L | 29 | 9 | 10 | 2 |
| CIMP-H | 21 | 14 | 3 | 0 |

**Figure S2. A LY6G6D mediated gene signature for Mucinous CRC. A)** Heatmap of differentially expressed genes according to mucinous versus Adenocarcinomas in rectal adenocarcinoma (READ) from TCGA database. **B)** Upper panel, GO pathways analysis of shared COAD and READ down-regulated genes in mucinous CRC. Lower panel, the down-regulated genes constitute a transcriptomic gene network selective of CRC involving secretion, solute transporter and immunity. **C)** Distribution of microsatellite instability (MSI), CpG island methylator phenotype (CIMP) and chromosomal instability (CIN) in mucinous vs non-mucinous CRC from TCGA database. **D)** Distribution of CIMP features in mucinous vs non-mucinous CRC from TCGA.

# Figure S3

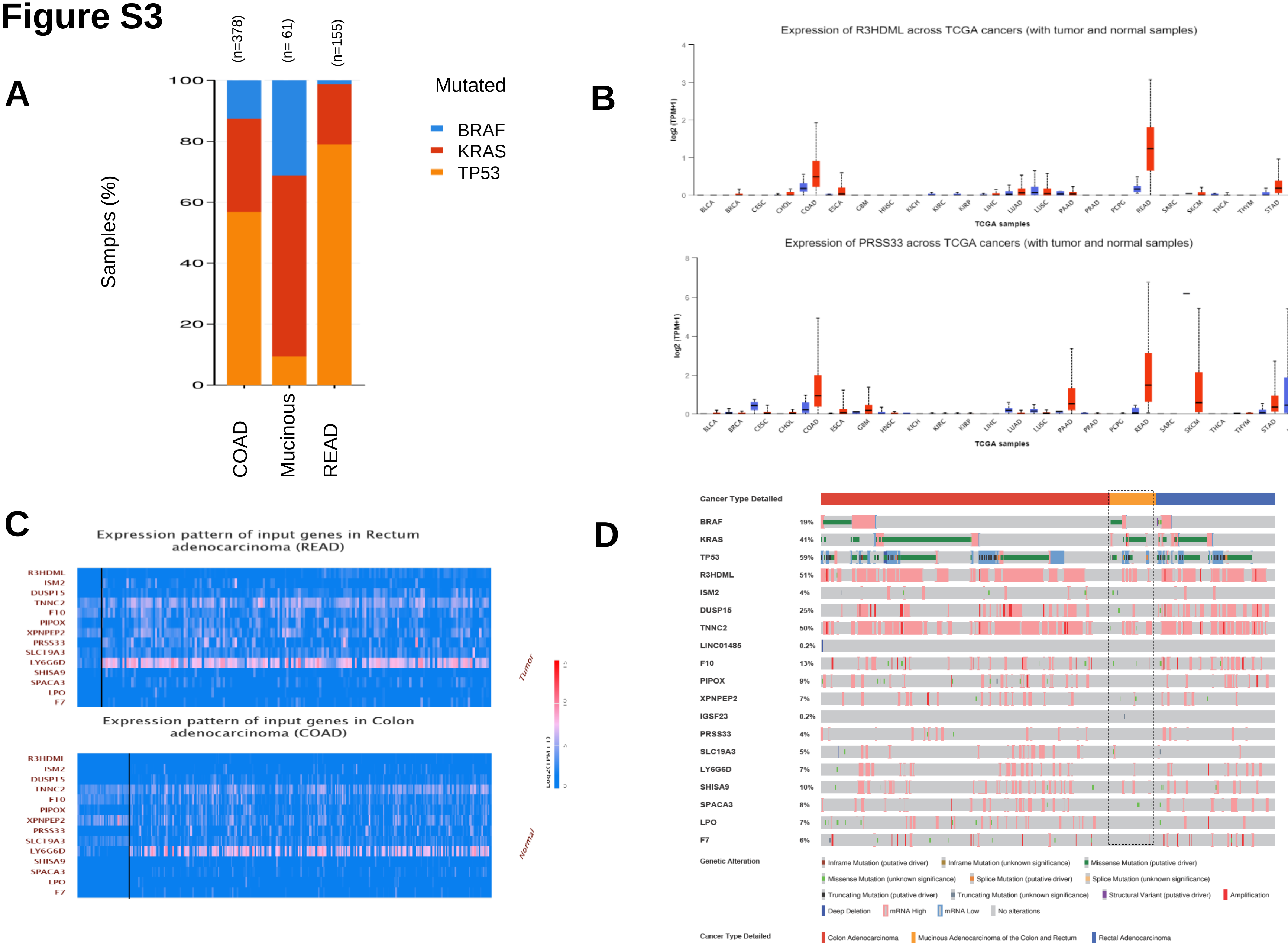


**Figure S3. Genomic alterations of Mucinous down-regulated gene signature in CRC. A)** Distribution of *BRAF, KRAS and p53* mutations in mucinous and non mucinous CRC from TCGA. **B)** Differential *R3HDML and PRSS33* mRNA expression across TCGA cancer types (red) using for comparison matched non-tumor tissues (blue). **C)** Heatmaps show the transcriptional profiling of Mucinous down-regulated gene signature in COAD and READ compared to normal mucosa. **D)** Genomic alterations of the genes down-regulated gene in Mucinous CRC, extrapolated from TCGA (cbioportal platform)..

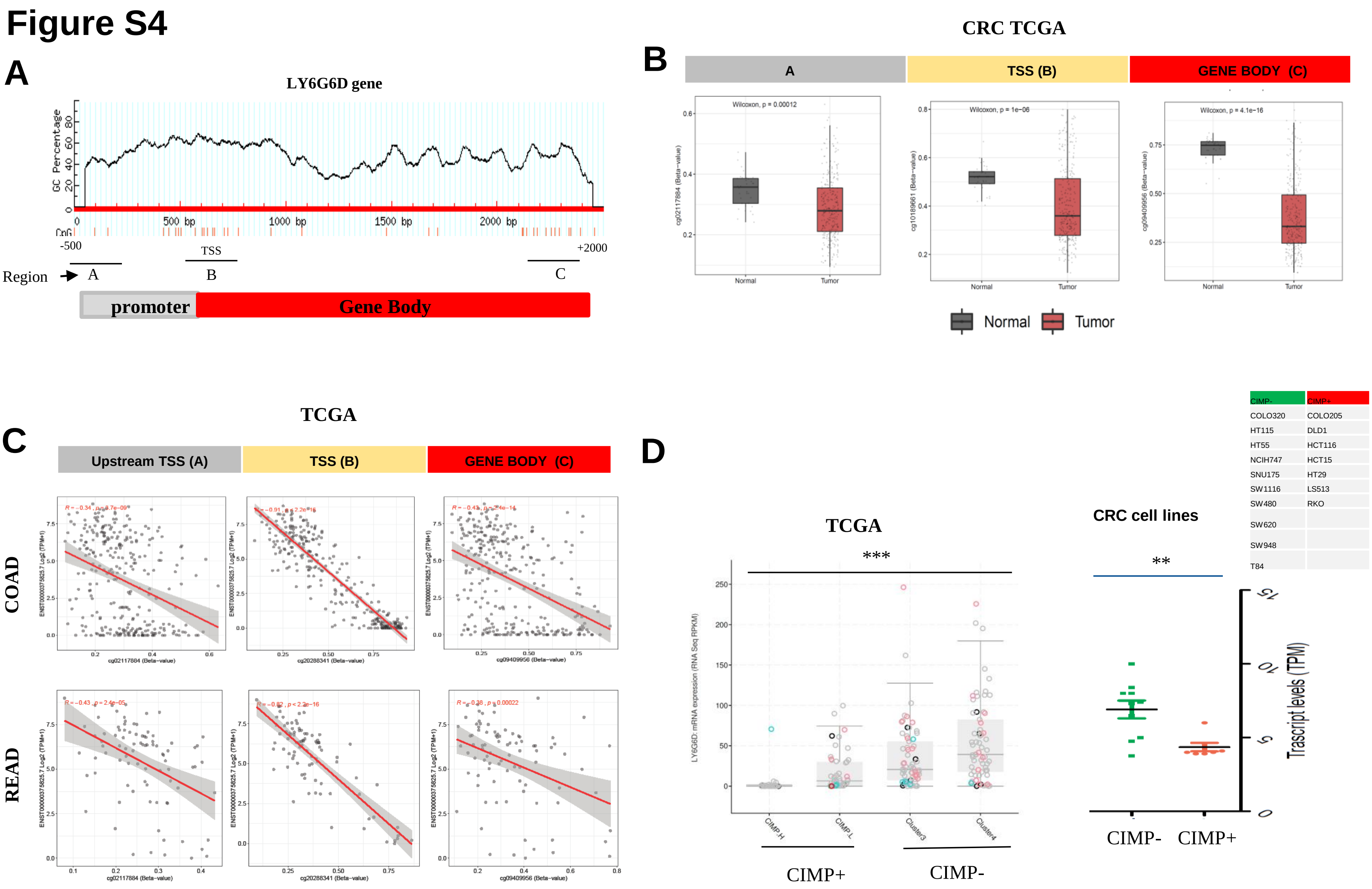


**Figure S4. DNA methylation changes in LY6G6D gene are intimately related with its transcript regulation. A)** The CpG island distribution encompassing the promoter region (-500 +1), transcription start site (TSS) and encoding region of LY6G6D gene up to +2000. CpG dinucleotides are represented as short and red vertical lines, whereas the position of investigated probes for DNA methylation is indicated as A (upstream TSS); B (overlapping TSS) and C (gene body). **B)** DNA Methylation changes in normal mucosa and CRC according to differential distribution on *LY6G6D* gene. **C)** Relation between *LY6G6D* transcript levels and DNA differential methylation on LY6G6D gene in COAD and READ from TCGA database. **D)** *LY6G6D* transcript levels in CIMP+ (CIMP-H and CIMP-low) versus CIMP- (cluster 3 and 4) in CRC samples and derived cell lines. ***$P\leq0.01$*; t test Welch-corrected.

# Figure S5

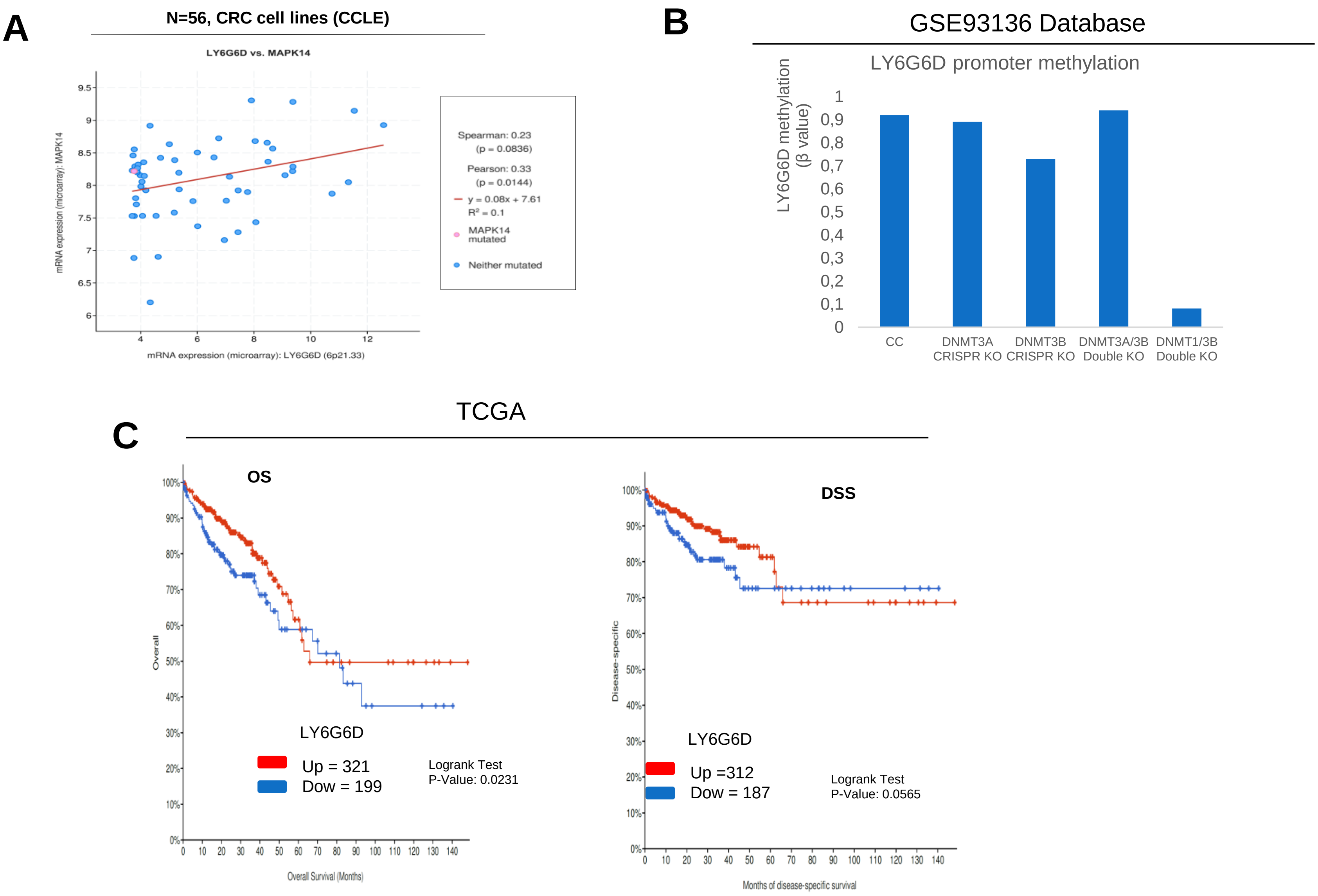


**Figure S5. Reduced expression of LY6G6D and its impact on CRC. A)** Relation between *LY6G6D and MAPK14* gene (p38α MAPK) transcript levels in CRC cell lines derived Cancer Cell Line Encyclopedia (CCLE). **B)** DNA methylation profile of *LY6G6D* promoter following genetic disruption of the indicated DNMTs in HCT116 and parental cells (CC). (β values: scores ranging between 0 and 1). **C)** Overall survival (OS) and disease specific survival (DSS) analysis in relation to the LY6G6D transcript levels extrapolated from TCGA data.

# Figure S6

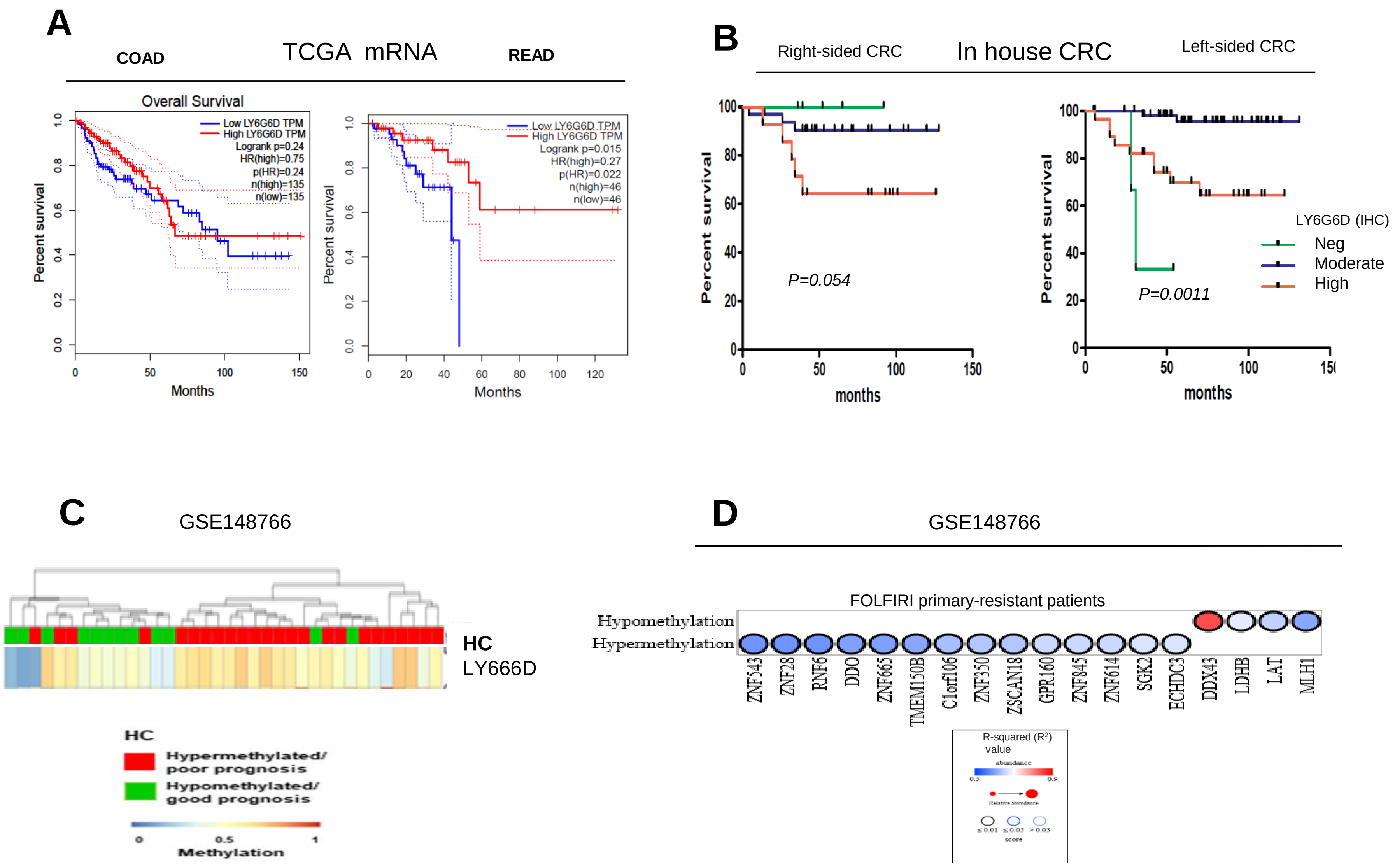


**Figure S6. LY6G6D expression predicts resistance to FOLFOX-based therapy in metastatic CRC patients. A)** Kaplan–Meier overall survival analysis in relation to LY6G6D transcript levels with Cutoff-High (>50%) and Cutoff-Low(<50%) in colon adenocarcinoma (COAD) and rectal adenocarcinoma (READ) from TCGA. **B)** LY6G6D IHC profile in relation to overall survival analysis in right-sided and left-sided CRC from our database. The *p-values* by log-rank test. **C)** Relationship between methylation profile of LY6G6D and DNA methylation prognostic groups in patients who received FOLFOX-based therapy derived from in house CRC GSE148766 database. **D)** The dot plot shows the top ranking hyper and hypo-methylated genes predicting resistance to FOLFIRI-based therapy, in which it is absent *LY6G6D*.
