# Supplementary material for: Lymphocyte antigen 6G6D-mediated modulation through p38α MAPK and DNA methylation in colorectal cancer
Source: Cancer Cell Int. 2022 Aug 11;22:253. doi: 10.1186/s12935-022-02672-1 (PMC9373545; doi:10.1186/s12935-022-02672-1)
Supplement: Supplementary file 2 — Additional file 2: Table S1. Differentially expressed genes Mucinous vs Adenocarcinoma in COAD. [file 12935_2022_2672_MOESM2_ESM.docx]

| **Table 1. DEGs Mucinous VS Adenocarcinoma in COAD** | | | | | | |
| --- | --- | --- | --- | --- | --- | --- |
| Genes | logFC | logCPM | LR | PValue | FDR | Status |
| KLRG2 | -1,5029562 | 0,39571777 | 13,1605392 | 0,00028591 | 0,00111145 | down |
| RIIAD1 | -1,5046878 | 0,20467758 | 24,3198797 | 8,16E-07 | 6,51E-06 | down |
| PKLR | -1,5065908 | 1,79322641 | 19,3613765 | 1,08E-05 | 6,30E-05 | down |
| OIT3 | -1,5073166 | 1,0527569 | 41,3783797 | 1,25E-10 | 2,58E-09 | down |
| FGB | -1,5092669 | 0,8678246 | 11,5576149 | 0,00067472 | 0,00234132 | down |
| NKD1 | -1,5097643 | 6,81946094 | 34,2096833 | 4,95E-09 | 7,01E-08 | down |
| NPTX2 | -1,5279216 | 4,70325474 | 23,5664858 | 1,21E-06 | 9,21E-06 | down |
| GLDC | -1,5288335 | 1,35961268 | 26,5969594 | 2,51E-07 | 2,30E-06 | down |
| GBX2 | -1,5296741 | -0,9651468 | 23,5862847 | 1,19E-06 | 9,13E-06 | down |
| SERPINA10 | -1,5359774 | 1,38614181 | 16,011904 | 6,29E-05 | 0,00029462 | down |
| ZNF556 | -1,5361551 | -0,1391148 | 10,339619 | 0,00130205 | 0,00411934 | down |
| PAK5 | -1,5410141 | -1,8758487 | 9,3104822 | 0,00227847 | 0,00666067 | down |
| GRK1 | -1,5415061 | -0,828729 | 22,6561328 | 1,94E-06 | 1,40E-05 | down |
| REEP1 | -1,5418475 | 3,06104262 | 38,704726 | 4,93E-10 | 8,78E-09 | down |
| TRIM54 | -1,5510474 | 1,71591678 | 16,3209236 | 5,35E-05 | 0,00025465 | down |
| RUBCNL | -1,571728 | 6,74366018 | 64,5237197 | 9,54E-16 | 6,96E-14 | down |
| R3HDML | -1,5826383 | 1,09443721 | 41,797812 | 1,01E-10 | 2,13E-09 | down |
| RUNDC3A | -1,5955446 | 0,66345334 | 31,4046003 | 2,09E-08 | 2,53E-07 | down |
| ISM2 | -1,6116126 | 1,41969769 | 19,3318947 | 1,10E-05 | 6,38E-05 | down |
| RGR | -1,6137706 | -0,8639401 | 12,2055283 | 0,00047648 | 0,00172881 | down |
| SLC22A31 | -1,6229614 | 1,16537574 | 15,1861497 | 9,74E-05 | 0,00043164 | down |
| FGGY | -1,6331497 | 5,46908009 | 56,5362185 | 5,52E-14 | 2,62E-12 | down |
| CAMKV | -1,6540364 | 0,57695819 | 19,2194139 | 1,17E-05 | 6,72E-05 | down |
| C1orf105 | -1,6898266 | -0,7321721 | 37,0408111 | 1,16E-09 | 1,89E-08 | down |
| SMTNL2 | -1,6933896 | 0,95869798 | 41,0307846 | 1,50E-10 | 3,03E-09 | down |
| CLDN9 | -1,6936877 | 1,22883791 | 52,5165082 | 4,27E-13 | 1,65E-11 | down |
| DUSP15 | -1,6960293 | 1,61180724 | 65,2722873 | 6,52E-16 | 4,80E-14 | down |
| HEPHL1 | -1,6990509 | 1,32521191 | 27,6915188 | 1,42E-07 | 1,40E-06 | down |
| CLDN6 | -1,7039883 | -0,9141361 | 16,0206826 | 6,27E-05 | 0,00029344 | down |
| GNG4 | -1,7083529 | 5,70486884 | 34,0930516 | 5,25E-09 | 7,37E-08 | down |
| SLC6A4 | -1,716551 | 2,1205104 | 31,2817855 | 2,23E-08 | 2,66E-07 | down |
| AC021683.2 | -1,7272425 | -0,5679001 | 22,7658615 | 1,83E-06 | 1,34E-05 | down |
| EREG | -1,7365523 | 6,04266605 | 43,2029652 | 4,93E-11 | 1,11E-09 | down |
| CPN1 | -1,7396647 | 0,00407132 | 14,0710608 | 0,00017603 | 0,00072547 | down |
| TNNC2 | -1,7505581 | 3,91070938 | 45,0911741 | 1,88E-11 | 4,73E-10 | down |
| CHRNB2 | -1,7528192 | 0,11313895 | 21,6572706 | 3,26E-06 | 2,22E-05 | down |
| SLC30A2 | -1,7642849 | 1,97917477 | 27,7799718 | 1,36E-07 | 1,34E-06 | down |
| LINC01485 | -1,7713976 | -0,4578217 | 26,5750665 | 2,53E-07 | 2,32E-06 | down |
| GLYATL2 | -1,7751428 | -1,3752054 | 28,5889612 | 8,95E-08 | 9,23E-07 | down |
| F10 | -1,7765144 | 2,70153942 | 58,6006073 | 1,93E-14 | 1,02E-12 | down |
| C10orf82 | -1,7813863 | 0,51795852 | 20,5388893 | 5,84E-06 | 3,70E-05 | down |
| MTTP | -1,7827573 | 3,12231097 | 17,2999204 | 3,19E-05 | 0,00016254 | down |
| PIPOX | -1,7885795 | 3,10711328 | 43,2757149 | 4,75E-11 | 1,07E-09 | down |
| XPNPEP2 | -1,8095613 | 3,87976027 | 39,4583069 | 3,35E-10 | 6,22E-09 | down |
| SERPINA7 | -1,8344322 | 0,37841363 | 15,129986 | 0,00010036 | 0,00044339 | down |
| PAH | -1,8503909 | 2,21065911 | 24,4333566 | 7,69E-07 | 6,18E-06 | down |
| GPR143 | -1,8598901 | 3,30325625 | 56,3615224 | 6,03E-14 | 2,86E-12 | down |
| PCSK1N | -1,8623805 | 2,50978851 | 22,7035298 | 1,89E-06 | 1,37E-05 | down |
| IGSF23 | -1,8631497 | -0,2331004 | 52,7545598 | 3,78E-13 | 1,47E-11 | down |
| EMX2 | -1,8758088 | -0,9111884 | 11,1804579 | 0,00082663 | 0,00278939 | down |
| TMEM252 | -1,8849095 | 1,64311056 | 27,1117438 | 1,92E-07 | 1,81E-06 | down |
| PCDH19 | -1,8877593 | 2,35863945 | 38,7913633 | 4,72E-10 | 8,44E-09 | down |
| CELF3 | -1,8971263 | 1,19150291 | 40,1090986 | 2,40E-10 | 4,64E-09 | down |
| CPLX2 | -1,9091129 | 2,1589952 | 36,0721535 | 1,90E-09 | 2,97E-08 | down |
| CLDN8 | -1,9276592 | 0,57184097 | 8,69191666 | 0,00319624 | 0,00893765 | down |
| KRT6C | -1,9465717 | -0,8765609 | 10,8858554 | 0,00096901 | 0,00319718 | down |
| PNLIPRP2 | -1,951248 | 1,75868838 | 34,1742987 | 5,04E-09 | 7,11E-08 | down |
| PRSS33 | -1,9573248 | 3,18324642 | 33,735526 | 6,31E-09 | 8,70E-08 | down |
| SLC19A3 | -1,9657141 | 3,48644634 | 71,6729286 | 2,54E-17 | 2,56E-15 | down |
| HSD52 | -1,9856354 | -1,0022259 | 23,8554028 | 1,04E-06 | 8,06E-06 | down |
| CYP24A1 | -1,9883278 | 0,56697275 | 31,051879 | 2,51E-08 | 2,97E-07 | down |
| LY6G6D | -1,9924486 | 3,27079218 | 22,8795289 | 1,72E-06 | 1,26E-05 | down |
| GPR83 | -1,9987446 | -0,1113907 | 23,7544017 | 1,09E-06 | 8,45E-06 | down |
| SPRR2A | -2,0351552 | 1,13454678 | 9,75064416 | 0,0017926 | 0,0054191 | down |
| SLC14A1 | -2,1047513 | 3,37220292 | 31,0151559 | 2,56E-08 | 3,02E-07 | down |
| MAP7D2 | -2,1152349 | 3,51206026 | 38,9154381 | 4,43E-10 | 7,98E-09 | down |
| SHISA9 | -2,1565688 | 1,57154663 | 18,0171088 | 2,19E-05 | 0,00011727 | down |
| CYP2W1 | -2,1728781 | 5,99220613 | 40,4929375 | 1,97E-10 | 3,88E-09 | down |
| LIPC | -2,2012757 | 0,49573523 | 32,8284435 | 1,01E-08 | 1,30E-07 | down |
| GLRA2 | -2,2043017 | 1,45826503 | 27,0337664 | 2,00E-07 | 1,88E-06 | down |
| FZD10 | -2,220592 | 2,81007521 | 35,3581753 | 2,74E-09 | 4,12E-08 | down |
| MYBPHL | -2,2349452 | -0,5861439 | 20,0453309 | 7,56E-06 | 4,64E-05 | down |
| KNG1 | -2,2424435 | -0,1804366 | 45,4060871 | 1,60E-11 | 4,11E-10 | down |
| SPACA3 | -2,2496235 | 0,12389161 | 38,0824423 | 6,78E-10 | 1,17E-08 | down |
| LPO | -2,2755309 | 0,11155775 | 41,5809469 | 1,13E-10 | 2,36E-09 | down |
| CEL | -2,2806674 | 5,33941497 | 41,9507497 | 9,36E-11 | 1,98E-09 | down |
| F7 | -2,2946432 | 1,1269445 | 41,2702631 | 1,33E-10 | 2,71E-09 | down |
| SYN3 | -2,3202283 | 1,65981055 | 47,6029289 | 5,22E-12 | 1,51E-10 | down |
| SLC13A3 | -2,3202551 | 3,44711033 | 54,0034691 | 2,00E-13 | 8,22E-12 | down |
| NOTUM | -2,3308857 | 6,46460494 | 37,858376 | 7,61E-10 | 1,30E-08 | down |
| TKTL1 | -2,3888664 | 0,94272727 | 11,5575702 | 0,00067474 | 0,00234132 | down |
| IGF2BP1 | -2,4001649 | 2,49305519 | 26,8921015 | 2,15E-07 | 2,00E-06 | down |
| VWA5B1 | -2,4006097 | -0,0673146 | 17,6321989 | 2,68E-05 | 0,00013997 | down |
| CHGB | -2,407957 | 3,79706459 | 24,68871 | 6,74E-07 | 5,51E-06 | down |
| CTNNA2 | -2,444024 | 2,08293894 | 25,0655455 | 5,54E-07 | 4,62E-06 | down |
| MAGEA11 | -2,4533088 | 1,02631081 | 9,66671104 | 0,00187637 | 0,0056371 | down |
| DRD2 | -2,4757383 | 2,11727749 | 46,1052192 | 1,12E-11 | 2,99E-10 | down |
| SLC22A11 | -2,4859333 | 1,59310195 | 51,7809881 | 6,20E-13 | 2,27E-11 | down |
| EYA1 | -2,4929435 | 1,5504194 | 41,3203041 | 1,29E-10 | 2,65E-09 | down |
| LINC01411 | -2,5188979 | 1,1357217 | 35,0830876 | 3,16E-09 | 4,69E-08 | down |
| SULT2A1 | -2,6487031 | 0,1747541 | 24,2386644 | 8,51E-07 | 6,76E-06 | down |
| PLA2G12B | -2,6681348 | 2,44184403 | 43,5518292 | 4,13E-11 | 9,44E-10 | down |
| KRT40 | -2,7720439 | 1,40021859 | 33,4255029 | 7,40E-09 | 9,94E-08 | down |
| PRSS56 | -2,7731503 | 2,85778139 | 11,8291622 | 0,0005831 | 0,00206239 | down |
| TMPRSS11E | -2,7808853 | 0,41276945 | 12,4443047 | 0,00041927 | 0,00154676 | down |
| TMEM132C | -2,7919299 | 1,28783682 | 28,2744216 | 1,05E-07 | 1,07E-06 | down |
| RBP2 | -2,7975979 | 2,69366973 | 65,8869147 | 4,78E-16 | 3,72E-14 | down |
| GRPR | -2,8004058 | 0,09664973 | 55,5874433 | 8,94E-14 | 4,05E-12 | down |
| PTF1A | -2,8412261 | -1,3767518 | 27,4791844 | 1,59E-07 | 1,54E-06 | down |
| AIRE | -2,8514436 | 0,11165113 | 36,3439567 | 1,65E-09 | 2,62E-08 | down |
| HSPB3 | -2,8521111 | -0,6787454 | 25,136787 | 5,34E-07 | 4,46E-06 | down |
| HS3ST4 | -3,3419129 | -0,3446607 | 23,1014452 | 1,54E-06 | 1,14E-05 | down |
| CRAT37 | -3,3824835 | -0,5050893 | 46,0124463 | 1,18E-11 | 3,11E-10 | down |
| PAEP | -3,4303238 | 3,05551907 | 23,8116452 | 1,06E-06 | 8,23E-06 | down |
| CCL25 | -3,6755794 | 2,20247919 | 27,7623122 | 1,37E-07 | 1,35E-06 | down |
| SOHLH1 | -3,7658861 | -1,1222514 | 18,8093838 | 1,44E-05 | 8,15E-05 | down |
| KRT14 | -3,9055818 | 2,10378118 | 18,3696329 | 1,82E-05 | 0,00010005 | down |
| MAGEB17 | -3,997815 | 1,66476688 | 51,3893336 | 7,57E-13 | 2,71E-11 | down |
| GDF10 | -4,0313462 | 1,17246005 | 45,6490424 | 1,41E-11 | 3,69E-10 | down |
| ORM1 | -4,5044513 | 2,20189789 | 33,3079795 | 7,87E-09 | 1,05E-07 | down |
| PPBP | -4,5532365 | 6,3667602 | 47,3803665 | 5,85E-12 | 1,65E-10 | down |
| SFTPC | -5,3698113 | 0,11230122 | 11,2168605 | 0,00081058 | 0,00274506 | down |
| UGT2B4 | 4,80472342 | -0,228884 | 102,767847 | 3,77E-24 | 1,16E-21 | up |
| NTS | 4,66210424 | 3,47834186 | 158,771188 | 2,10E-36 | 3,55E-33 | up |
| ERVH48-1 | 4,03467101 | -0,3674151 | 272,135772 | 3,89E-61 | 2,19E-57 | up |
| COLEC10 | 3,94532373 | -0,6340012 | 402,48256 | 1,59E-89 | 2,69E-85 | up |
| GCG | 3,73546517 | 2,66240035 | 84,4498645 | 3,94E-20 | 6,74E-18 | up |
| FGL1 | 3,58022269 | -0,4406376 | 69,2339053 | 8,75E-17 | 7,75E-15 | up |
| FBXO40 | 3,5703964 | -1,0430689 | 56,2379542 | 6,42E-14 | 3,01E-12 | up |
| NNAT | 3,45889816 | 2,53948325 | 338,908819 | 1,10E-75 | 9,33E-72 | up |
| SYNGR4 | 3,42617909 | 0,2788848 | 217,284623 | 3,54E-49 | 1,50E-45 | up |
| CALCA | 3,34496465 | 1,61937303 | 100,307567 | 1,30E-23 | 3,56E-21 | up |
| RNU5E-1 | 3,20317021 | 1,67805522 | 26,2603426 | 2,98E-07 | 2,66E-06 | up |
| MSMB | 2,99674423 | -0,8507019 | 28,7370617 | 8,29E-08 | 8,63E-07 | up |
| CNGA3 | 2,98403176 | -0,6181565 | 111,131023 | 5,54E-26 | 2,08E-23 | up |
| PROK2 | 2,9816489 | 0,38999168 | 133,110196 | 8,55E-31 | 6,29E-28 | up |
| H3C12 | 2,95553609 | 1,4985403 | 54,9525181 | 1,23E-13 | 5,40E-12 | up |
| H2AC21 | 2,93084791 | 1,41105856 | 25,3734074 | 4,72E-07 | 4,01E-06 | up |
| RNU6ATAC | 2,80322306 | 0,23933264 | 22,6228799 | 1,97E-06 | 1,42E-05 | up |
| MUCL3 | 2,68416524 | -0,2596357 | 46,603392 | 8,69E-12 | 2,37E-10 | up |
| H4C1 | 2,62575399 | -0,1638058 | 27,5190632 | 1,56E-07 | 1,51E-06 | up |
| DBH | 2,57403925 | -0,5705033 | 128,4189 | 9,09E-30 | 5,92E-27 | up |
| ALOX15B | 2,56138497 | 1,49258977 | 182,506115 | 1,37E-41 | 3,32E-38 | up |
| DGKB | 2,55624245 | 0,43003246 | 67,3827948 | 2,24E-16 | 1,85E-14 | up |
| LEP | 2,55264993 | -0,6265504 | 32,9997977 | 9,22E-09 | 1,21E-07 | up |
| TFF2 | 2,52481835 | 4,44720369 | 74,2128402 | 7,01E-18 | 7,86E-16 | up |
| H2AC14 | 2,52184732 | 2,33542696 | 24,7466163 | 6,54E-07 | 5,36E-06 | up |
| SST | 2,51833979 | -0,9502199 | 55,6941661 | 8,47E-14 | 3,88E-12 | up |
| LUCAT1 | 2,50937854 | 0,01627599 | 149,572282 | 2,15E-34 | 2,27E-31 | up |
| H3C11 | 2,49941698 | 1,45250164 | 20,4127749 | 6,24E-06 | 3,93E-05 | up |
| ELOVL2 | 2,49902486 | -0,2994753 | 154,97362 | 1,42E-35 | 2,18E-32 | up |
| H4C3 | 2,47092951 | 2,39203639 | 27,0688556 | 1,96E-07 | 1,85E-06 | up |
| H1-3 | 2,46111188 | 2,4497045 | 33,7016796 | 6,42E-09 | 8,83E-08 | up |
| H4C4 | 2,45004744 | 2,61093222 | 27,2721962 | 1,77E-07 | 1,68E-06 | up |
| HCAR2 | 2,4256056 | 1,90470677 | 112,432148 | 2,87E-26 | 1,16E-23 | up |
| SLITRK4 | 2,39995871 | -1,1231261 | 94,1830097 | 2,88E-22 | 6,41E-20 | up |
| FCAR | 2,3520688 | -0,2842586 | 121,574503 | 2,86E-28 | 1,56E-25 | up |
| H2BC17 | 2,34815104 | 1,95452362 | 29,1881586 | 6,57E-08 | 7,04E-07 | up |
| CXCL5 | 2,32501586 | 5,47065814 | 84,3985254 | 4,04E-20 | 6,85E-18 | up |
| H2AC13 | 2,3144134 | 2,42990213 | 43,1752269 | 5,01E-11 | 1,12E-09 | up |
| H2AC7 | 2,28999491 | 2,94078329 | 49,2579341 | 2,24E-12 | 7,10E-11 | up |
| KIF19 | 2,28219316 | 1,85475792 | 107,723911 | 3,09E-25 | 1,05E-22 | up |
| H2AC4 | 2,27154717 | 0,97810015 | 16,4845046 | 4,90E-05 | 0,00023639 | up |
| H2AC20 | 2,27022104 | 2,97003431 | 63,5362461 | 1,57E-15 | 1,10E-13 | up |
| CCDC144A | 2,26625653 | -1,2882916 | 43,9982341 | 3,29E-11 | 7,75E-10 | up |
| SCARNA21 | 2,26288172 | 1,97694482 | 15,6864733 | 7,48E-05 | 0,00034291 | up |
| H3C13 | 2,25738456 | -0,653838 | 48,5057045 | 3,29E-12 | 9,97E-11 | up |
| H2BC13 | 2,2547491 | 1,3355176 | 40,0247275 | 2,51E-10 | 4,80E-09 | up |
| FPR2 | 2,23540458 | 1,35888059 | 112,519167 | 2,75E-26 | 1,14E-23 | up |
| SPINK4 | 2,21108427 | 7,03234668 | 71,2843255 | 3,09E-17 | 2,99E-15 | up |
| H2BC14 | 2,17999492 | 0,23007619 | 12,6534322 | 0,00037488 | 0,00140535 | up |
| H2BC10 | 2,17329435 | 1,50329471 | 10,4408794 | 0,00123257 | 0,00393853 | up |
| PON1 | 2,16319423 | -0,8344614 | 38,9452522 | 4,36E-10 | 7,86E-09 | up |
| H3C1 | 2,16231245 | 0,41121681 | 19,9285998 | 8,04E-06 | 4,89E-05 | up |
| AQP3 | 2,1455356 | 5,51974095 | 149,730016 | 1,99E-34 | 2,24E-31 | up |
| FAM177B | 2,13017942 | 1,46492691 | 96,6326708 | 8,35E-23 | 2,02E-20 | up |
| OSM | 2,1079218 | 3,0926632 | 150,984364 | 1,06E-34 | 1,38E-31 | up |
| PADI3 | 2,10554853 | 2,37048807 | 40,5796329 | 1,89E-10 | 3,74E-09 | up |
| NEFL | 2,09532384 | 0,17445924 | 48,2736881 | 3,71E-12 | 1,11E-10 | up |
| CPS1 | 2,09439298 | 4,47371831 | 53,0852152 | 3,19E-13 | 1,27E-11 | up |
| KCNJ15 | 2,08023024 | 1,27431494 | 147,172832 | 7,19E-34 | 7,16E-31 | up |
| EPYC | 2,06697795 | 1,4039978 | 39,8909926 | 2,69E-10 | 5,10E-09 | up |
| TRARG1 | 2,05816131 | -0,2405467 | 10,1107318 | 0,00147408 | 0,00458141 | up |
| CSF3R | 2,05418024 | 3,70065082 | 165,824836 | 6,04E-38 | 1,28E-34 | up |
| MUC2 | 2,04929225 | 10,1803744 | 77,8342754 | 1,12E-18 | 1,45E-16 | up |
| CD300E | 2,04363228 | 1,37469702 | 139,882239 | 2,82E-32 | 2,39E-29 | up |
| B3GNT6 | 2,03658963 | 3,95834556 | 54,3010078 | 1,72E-13 | 7,24E-12 | up |
| H4C2 | 2,03465195 | 1,4302957 | 16,4345615 | 5,04E-05 | 0,00024201 | up |
| H1-4 | 2,02787832 | 4,29333467 | 26,3848528 | 2,80E-07 | 2,52E-06 | up |
| IGFALS | 2,02330043 | 1,53068758 | 51,5886513 | 6,84E-13 | 2,47E-11 | up |
| HSPA2 | 2,0209849 | 4,80229443 | 200,286231 | 1,81E-45 | 6,12E-42 | up |
| MYCN | 2,01938069 | 2,38737229 | 118,865895 | 1,12E-27 | 5,58E-25 | up |
| OPRD1 | 2,01420006 | 0,9562643 | 65,3112754 | 6,40E-16 | 4,73E-14 | up |
| H2AC17 | 2,01299699 | 1,47759457 | 31,8596557 | 1,66E-08 | 2,05E-07 | up |
| SEZ6L | 2,00646397 | -1,1177026 | 45,6518921 | 1,41E-11 | 3,69E-10 | up |
| ZNF385B | 2,00198153 | -0,9225403 | 75,4734862 | 3,70E-18 | 4,29E-16 | up |
| IL1B | 1,99966472 | 5,05750909 | 150,447948 | 1,38E-34 | 1,67E-31 | up |
| AQP9 | 1,99118615 | 2,95780464 | 111,575738 | 4,43E-26 | 1,74E-23 | up |
| H2BC11 | 1,99087241 | 2,74748701 | 68,1131934 | 1,54E-16 | 1,33E-14 | up |
| ALDH1A2 | 1,99035291 | 2,27329168 | 54,9965546 | 1,21E-13 | 5,29E-12 | up |
| PLIN4 | 1,98744793 | 3,897601 | 52,8371078 | 3,62E-13 | 1,43E-11 | up |
| RNU4-1 | 1,98392241 | 3,42552896 | 12,3436915 | 0,00044248 | 0,00161933 | up |
| SIGLEC5 | 1,9807635 | -1,0648105 | 128,173376 | 1,03E-29 | 6,45E-27 | up |
| RHBDL3 | 1,95943834 | 0,36915881 | 79,7247252 | 4,30E-19 | 5,83E-17 | up |
| GAS1 | 1,95399563 | 3,15693926 | 97,9649425 | 4,26E-23 | 1,06E-20 | up |
| SLC16A7 | 1,94942042 | 2,16979325 | 135,653077 | 2,38E-31 | 1,83E-28 | up |
| H1-5 | 1,94099977 | 4,17749293 | 19,4913842 | 1,01E-05 | 5,95E-05 | up |
| LINC02649 | 1,93160141 | -0,0702611 | 93,5870379 | 3,89E-22 | 8,44E-20 | up |
| MRTFA-AS1 | 1,92858962 | -0,93621 | 26,4569835 | 2,69E-07 | 2,44E-06 | up |
| CXCL8 | 1,92111313 | 7,37861451 | 101,129214 | 8,62E-24 | 2,43E-21 | up |
| SCEL | 1,91712286 | 2,18206722 | 50,9632207 | 9,41E-13 | 3,28E-11 | up |
| CYP4F8 | 1,91058038 | -0,4540905 | 49,7512515 | 1,75E-12 | 5,65E-11 | up |
| H2BC4 | 1,91017927 | 2,66600989 | 56,2918145 | 6,25E-14 | 2,94E-12 | up |
| ITLN2 | 1,89014157 | 0,76563346 | 20,8877535 | 4,87E-06 | 3,16E-05 | up |
| BCAT1 | 1,87792604 | 4,1061172 | 163,462955 | 1,98E-37 | 3,73E-34 | up |
| CXCR1 | 1,87519338 | 0,77123433 | 78,9002173 | 6,53E-19 | 8,64E-17 | up |
| G0S2 | 1,86272969 | 4,31478096 | 154,66401 | 1,66E-35 | 2,34E-32 | up |
| CXCR2 | 1,8462735 | 1,44666276 | 105,311242 | 1,04E-24 | 3,33E-22 | up |
| SPDEF | 1,84623083 | 4,89326593 | 97,9965004 | 4,19E-23 | 1,06E-20 | up |
| H3C4 | 1,84060005 | 1,67330388 | 53,4986787 | 2,59E-13 | 1,05E-11 | up |
| RASD1 | 1,82801204 | 3,84507441 | 120,549687 | 4,80E-28 | 2,54E-25 | up |
| H4C8 | 1,82302464 | 1,98902232 | 52,5526634 | 4,19E-13 | 1,62E-11 | up |
| PLEKHG4B | 1,8120646 | -0,9481708 | 59,2247863 | 1,41E-14 | 7,63E-13 | up |
| ADGRG3 | 1,8076725 | 1,22674993 | 132,17879 | 1,37E-30 | 9,64E-28 | up |
| ABCA4 | 1,80643612 | -0,3467114 | 66,596945 | 3,33E-16 | 2,65E-14 | up |
| FFAR2 | 1,80218842 | 1,58535417 | 115,31594 | 6,71E-27 | 2,99E-24 | up |
| REP15 | 1,78679032 | 1,00174045 | 60,8387789 | 6,19E-15 | 3,68E-13 | up |
| CBFA2T3 | 1,78623415 | 3,30695244 | 103,446173 | 2,68E-24 | 8,39E-22 | up |
| ITGAD | 1,77937208 | -1,4798632 | 88,7874218 | 4,40E-21 | 8,55E-19 | up |
| LINC00261 | 1,77518116 | 5,66481419 | 71,5230887 | 2,74E-17 | 2,73E-15 | up |
| H2AC12 | 1,76833438 | 1,55046336 | 12,0117117 | 0,00052867 | 0,00189463 | up |
| CYP3A4 | 1,7677853 | 0,56383883 | 43,8369905 | 3,57E-11 | 8,29E-10 | up |
| MEFV | 1,76755825 | 0,34370298 | 108,367017 | 2,23E-25 | 7,71E-23 | up |
| DIRAS1 | 1,76157258 | 0,26284854 | 83,2333153 | 7,29E-20 | 1,20E-17 | up |
| HCAR3 | 1,76000689 | 0,30267004 | 38,17625 | 6,46E-10 | 1,12E-08 | up |
| VGLL3 | 1,75926707 | 2,98058396 | 95,6738933 | 1,35E-22 | 3,10E-20 | up |
| FCGR3B | 1,75850582 | 2,34111551 | 66,9066343 | 2,85E-16 | 2,29E-14 | up |
| REG4 | 1,74899819 | 9,48248398 | 37,4392471 | 9,43E-10 | 1,57E-08 | up |
| CACNA1B | 1,74519494 | -0,7879054 | 36,1211715 | 1,85E-09 | 2,90E-08 | up |
| RORB | 1,74182584 | -1,6266975 | 73,3609216 | 1,08E-17 | 1,18E-15 | up |
| CNTN1 | 1,7394315 | 0,46861945 | 56,9931073 | 4,37E-14 | 2,14E-12 | up |
| USP32P1 | 1,73417456 | -1,0078881 | 32,5319679 | 1,17E-08 | 1,49E-07 | up |
| KLK5 | 1,73139001 | -1,3684044 | 22,7691484 | 1,83E-06 | 1,34E-05 | up |
| S100A12 | 1,7291894 | -0,9312617 | 52,2449653 | 4,90E-13 | 1,85E-11 | up |
| GALNT8 | 1,72354663 | 3,56547562 | 82,2787864 | 1,18E-19 | 1,87E-17 | up |
| TPH1 | 1,72112169 | 0,42231073 | 80,6539905 | 2,69E-19 | 3,82E-17 | up |
| MIR133A1HG | 1,71908388 | -1,3431018 | 12,8152869 | 0,0003438 | 0,00130708 | up |
| KIRREL3 | 1,71633133 | -0,5836033 | 90,2713139 | 2,08E-21 | 4,18E-19 | up |
| BEX1 | 1,71496884 | -1,0663278 | 40,9863699 | 1,53E-10 | 3,09E-09 | up |
| SHISA3 | 1,70929639 | 0,57368398 | 75,6890476 | 3,32E-18 | 3,93E-16 | up |
| H2BU1 | 1,70888668 | 0,09139447 | 77,0610794 | 1,66E-18 | 2,05E-16 | up |
| CCK | 1,70470032 | -0,6142072 | 12,334733 | 0,00044461 | 0,00162571 | up |
| RAB3B | 1,70423527 | 2,95618355 | 84,7936178 | 3,31E-20 | 5,72E-18 | up |
| LBP | 1,69751294 | 0,24838825 | 37,7734173 | 7,95E-10 | 1,35E-08 | up |
| H2BC18 | 1,69609358 | 1,25625246 | 49,8018233 | 1,70E-12 | 5,54E-11 | up |
| KLK3 | 1,68598184 | 0,32198803 | 42,5877748 | 6,76E-11 | 1,47E-09 | up |
| ADAM12 | 1,68229491 | 5,1241555 | 89,0464276 | 3,86E-21 | 7,59E-19 | up |
| HRH2 | 1,6817183 | -0,2594919 | 77,0837995 | 1,64E-18 | 2,04E-16 | up |
| PRSS2 | 1,6807313 | 5,68939055 | 19,7666702 | 8,75E-06 | 5,27E-05 | up |
| MUC5AC | 1,67945246 | 6,2582137 | 28,7310238 | 8,32E-08 | 8,65E-07 | up |
| CSF2RB | 1,67855558 | 4,12188926 | 125,961135 | 3,14E-29 | 1,83E-26 | up |
| ITGAX | 1,67725986 | 4,5033362 | 140,450639 | 2,12E-32 | 1,89E-29 | up |
| SLC26A9 | 1,67110146 | 1,52455994 | 17,4469683 | 2,95E-05 | 0,00015219 | up |
| CD300LG | 1,66824027 | -1,9806418 | 25,3973036 | 4,67E-07 | 3,97E-06 | up |
| CYP1B1 | 1,66586319 | 3,2542317 | 61,2527071 | 5,02E-15 | 3,05E-13 | up |
| SLC11A1 | 1,66246668 | 3,60676185 | 131,999663 | 1,50E-30 | 1,01E-27 | up |
| RN7SL2 | 1,66113502 | 8,63920581 | 28,6422784 | 8,71E-08 | 9,00E-07 | up |
| MGAM | 1,66042261 | 0,48254762 | 91,0624229 | 1,39E-21 | 2,95E-19 | up |
| HS3ST3B1 | 1,65116413 | 2,1145063 | 183,430337 | 8,64E-42 | 2,44E-38 | up |
| BMPR1B | 1,64901279 | 0,11145237 | 52,1278105 | 5,20E-13 | 1,96E-11 | up |
| IGFL3 | 1,64717752 | -1,258895 | 34,254108 | 4,84E-09 | 6,88E-08 | up |
| ADAMTSL4-AS1 | 1,64660133 | 0,09830334 | 44,2504101 | 2,89E-11 | 6,91E-10 | up |
| AVPR1A | 1,64631793 | 0,95631704 | 110,406168 | 7,98E-26 | 2,94E-23 | up |
| BX322234.1 | 1,64473952 | -0,5940805 | 120,394006 | 5,19E-28 | 2,66E-25 | up |
| TREM1 | 1,6420141 | 2,537741 | 87,0129503 | 1,08E-20 | 2,05E-18 | up |
| H4C12 | 1,64188333 | -0,9703949 | 33,2507152 | 8,10E-09 | 1,08E-07 | up |
| FER1L6 | 1,64011591 | 2,61252768 | 45,0687131 | 1,90E-11 | 4,78E-10 | up |
| INSL4 | 1,63631369 | -1,0479503 | 11,9597131 | 0,00054363 | 0,0019392 | up |
| M1AP | 1,63353604 | -0,8609252 | 42,4043763 | 7,42E-11 | 1,60E-09 | up |
| KRT7 | 1,63114077 | 3,91348245 | 61,2525745 | 5,02E-15 | 3,05E-13 | up |
| CALB1 | 1,63019393 | 3,88452469 | 17,2870584 | 3,21E-05 | 0,00016345 | up |
| FPR1 | 1,62749204 | 3,08963121 | 98,0009162 | 4,18E-23 | 1,06E-20 | up |
| RHOQ-AS1 | 1,62353177 | -0,985975 | 45,0433898 | 1,93E-11 | 4,83E-10 | up |
| PEG10 | 1,6180049 | 3,2065991 | 36,3695859 | 1,63E-09 | 2,58E-08 | up |
| AMPH | 1,61733786 | 0,57364548 | 142,524634 | 7,47E-33 | 7,02E-30 | up |
| ERVFRD-1 | 1,61314431 | -1,4962179 | 28,8545647 | 7,80E-08 | 8,18E-07 | up |
| UNC5B-AS1 | 1,60964118 | -0,6066756 | 45,2466062 | 1,74E-11 | 4,44E-10 | up |
| CLDN18 | 1,60735665 | 5,03891303 | 13,6222453 | 0,00022352 | 0,0008956 | up |
| PLAAT5 | 1,60559949 | -0,1716394 | 38,5309168 | 5,39E-10 | 9,56E-09 | up |
| DSEL | 1,5988515 | 1,09085261 | 98,343965 | 3,52E-23 | 9,16E-21 | up |
| GFAP | 1,59715143 | -1,1471238 | 63,7513805 | 1,41E-15 | 9,91E-14 | up |
| SLAIN1 | 1,59525366 | 1,64777381 | 76,4924259 | 2,21E-18 | 2,69E-16 | up |
| TCN1 | 1,59490751 | 5,00420859 | 30,9976079 | 2,58E-08 | 3,04E-07 | up |
| REG1A | 1,5926619 | 9,07549103 | 22,5492535 | 2,05E-06 | 1,48E-05 | up |
| DOCK8-AS1 | 1,58438519 | -1,0554781 | 69,960226 | 6,05E-17 | 5,45E-15 | up |
| LINC01550 | 1,57865486 | -0,8011261 | 56,3381319 | 6,10E-14 | 2,89E-12 | up |
| COL6A5 | 1,57865264 | -1,8308825 | 39,4764801 | 3,32E-10 | 6,17E-09 | up |
| SIX3 | 1,57162705 | -1,2465087 | 20,6154001 | 5,61E-06 | 3,58E-05 | up |
| ANXA10 | 1,55822726 | 1,92680569 | 10,2027483 | 0,00140232 | 0,0043906 | up |
| ADAMTSL4 | 1,5559385 | 3,71833741 | 95,2798377 | 1,65E-22 | 3,73E-20 | up |
| FABP4 | 1,55552509 | 3,15996528 | 23,1345042 | 1,51E-06 | 1,12E-05 | up |
| CFAP46 | 1,55326876 | -0,8886275 | 32,6042489 | 1,13E-08 | 1,44E-07 | up |
| PRRG3 | 1,54290318 | -0,5748275 | 72,6388703 | 1,56E-17 | 1,63E-15 | up |
| IL17REL | 1,54053386 | -0,941703 | 39,2576137 | 3,71E-10 | 6,81E-09 | up |
| FCGBP | 1,53889711 | 9,80670526 | 43,6010667 | 4,03E-11 | 9,25E-10 | up |
| CCBE1 | 1,53746767 | 0,33012868 | 47,5033481 | 5,49E-12 | 1,57E-10 | up |
| CD70 | 1,53680172 | 1,2384209 | 63,2782737 | 1,79E-15 | 1,24E-13 | up |
| S100A8 | 1,53580555 | 3,09723454 | 60,9055737 | 5,99E-15 | 3,58E-13 | up |
| CP | 1,53436424 | 2,34788105 | 47,4076865 | 5,77E-12 | 1,64E-10 | up |
| MUC5B | 1,53325703 | 8,76629048 | 41,1288221 | 1,43E-10 | 2,90E-09 | up |
| ANO7 | 1,52956246 | 3,6847048 | 77,100414 | 1,62E-18 | 2,04E-16 | up |
| TEX41 | 1,52820355 | -1,4460408 | 50,2896071 | 1,33E-12 | 4,45E-11 | up |
| RAB26 | 1,52812844 | 2,47559071 | 72,8557551 | 1,39E-17 | 1,48E-15 | up |
| FAM181B | 1,5265215 | -1,8637655 | 37,5276139 | 9,01E-10 | 1,51E-08 | up |
| RNU4-2 | 1,52439154 | 4,98439098 | 8,62918067 | 0,0033082 | 0,0092051 | up |
| CLEC5A | 1,52387129 | 1,9937214 | 86,9264131 | 1,13E-20 | 2,12E-18 | up |
| FCGR2C | 1,52239855 | 0,55460225 | 80,9127868 | 2,36E-19 | 3,44E-17 | up |
| ITPK1-AS1 | 1,52133247 | -0,9419669 | 16,2998289 | 5,41E-05 | 0,00025714 | up |
| PTGS2 | 1,52125934 | 4,40029388 | 67,0662294 | 2,63E-16 | 2,15E-14 | up |
| CNDP1 | 1,51670907 | -0,6036092 | 24,3049694 | 8,22E-07 | 6,55E-06 | up |
| MMP13 | 1,51611643 | 1,36551382 | 36,2402636 | 1,74E-09 | 2,75E-08 | up |
| STEAP4 | 1,51453251 | 2,72506622 | 85,0879451 | 2,85E-20 | 4,98E-18 | up |
| H2BC7 | 1,50525198 | 0,61665657 | 17,5542506 | 2,79E-05 | 0,00014502 | up |
| COL24A1 | 1,5025507 | 1,32315478 | 77,4375604 | 1,37E-18 | 1,74E-16 | up |
| H1-2 | 1,5010361 | 5,13473328 | 54,9068031 | 1,26E-13 | 5,51E-12 | up |
| NRK | 1,50061862 | 0,05674508 | 38,0939917 | 6,74E-10 | 1,17E-08 | up |
| PRND | 1,50029959 | -0,6802879 | 40,5339662 | 1,93E-10 | 3,81E-09 | up |
