# Supplementary material for: Lymphocyte antigen 6G6D-mediated modulation through p38α MAPK and DNA methylation in colorectal cancer
Source: Cancer Cell Int. 2022 Aug 11;22:253. doi: 10.1186/s12935-022-02672-1 (PMC9373545; doi:10.1186/s12935-022-02672-1)
Supplement: Supplementary file 3 — Additional file 3: Table S2. Differentially expressed genes Mucinous vs Adenocarcinoma in READ. [file 12935_2022_2672_MOESM3_ESM.docx]

| **Table 2. DEGs Mucinous VS Adenocarcinoma in READ** | | | | | | |
| --- | --- | --- | --- | --- | --- | --- |
| Genes | logFC | logCPM | LR | PValue | FDR | Status |
| SLC51B | -1,50633 | 2,667991 | 14,10004 | 0,000173 | 0,004958 | down |
| TMEM63C | -1,56228 | 3,19582 | 12,78831 | 0,000349 | 0,008453 | down |
| LINC01558 | -1,5673 | 2,386283 | 14,52372 | 0,000138 | 0,004157 | down |
| ADGRF4 | -1,56794 | 3,393382 | 17,44597 | 2,96E-05 | 0,00124 | down |
| TH | -1,60481 | 1,107994 | 12,96378 | 0,000318 | 0,007843 | down |
| CCDC175 | -1,62272 | -0,87531 | 13,48136 | 0,000241 | 0,006398 | down |
| IGSF23 | -1,6543 | 0,014045 | 15,73152 | 7,30E-05 | 0,002542 | down |
| MIR3150BHG | -1,66005 | -1,91368 | 12,53802 | 0,000399 | 0,009315 | down |
| F10 | -1,67048 | 3,249248 | 12,68641 | 0,000368 | 0,008785 | down |
| LINC00514 | -1,69912 | -0,48701 | 13,38272 | 0,000254 | 0,006653 | down |
| TNNC2 | -1,74113 | 4,324267 | 12,94326 | 0,000321 | 0,007918 | down |
| LINC00525 | -1,74409 | 0,176805 | 17,17815 | 3,40E-05 | 0,001376 | down |
| DUSP15 | -1,78142 | 2,089018 | 14,71506 | 0,000125 | 0,003873 | down |
| R3HDML | -1,87856 | 1,160745 | 24,79865 | 6,36E-07 | 5,05E-05 | down |
| SSUH2 | -1,90514 | 2,467659 | 17,5432 | 2,81E-05 | 0,001193 | down |
| SLC19A3 | -2,01635 | 4,116514 | 21,65487 | 3,26E-06 | 0,000202 | down |
| HOXD10 | -2,01728 | 2,330698 | 14,48679 | 0,000141 | 0,004232 | down |
| UNC93A | -2,10203 | 2,50944 | 12,75969 | 0,000354 | 0,008534 | down |
| ST8SIA6 | -2,15097 | 0,066142 | 15,56257 | 7,98E-05 | 0,002739 | down |
| KIF6 | -2,17478 | -0,66952 | 14,57827 | 0,000134 | 0,004067 | down |
| HOXD11 | -2,17782 | 1,389726 | 13,94864 | 0,000188 | 0,005293 | down |
| ABCC2 | -2,20998 | 3,188409 | 14,34442 | 0,000152 | 0,004492 | down |
| XPNPEP2 | -2,25422 | 4,460877 | 16,47098 | 4,94E-05 | 0,00188 | down |
| SLC26A3 | -2,31478 | 8,060778 | 13,18185 | 0,000283 | 0,007258 | down |
| WNT11 | -2,52002 | 5,235038 | 19,73525 | 8,89E-06 | 0,000462 | down |
| CACNG4 | -2,59132 | 2,855063 | 12,40317 | 0,000429 | 0,009821 | down |
| LY6G6D | -2,71282 | 3,720539 | 20,06016 | 7,50E-06 | 0,000401 | down |
| LINC01485 | -2,83595 | -0,22606 | 14,4519 | 0,000144 | 0,00429 | down |
| CYP4F8 | -2,99204 | -0,17566 | 12,99837 | 0,000312 | 0,007741 | down |
| KCNT1 | -3,01319 | 0,119593 | 19,41034 | 1,05E-05 | 0,000529 | down |
| PRSS33 | -3,11834 | 4,153967 | 14,68466 | 0,000127 | 0,003922 | down |
| ACTL8 | -3,14899 | 1,638723 | 13,07101 | 0,0003 | 0,007551 | down |
| ERICH4 | -3,17181 | -1,41275 | 14,58339 | 0,000134 | 0,004064 | down |
| PIPOX | -3,30219 | 3,449549 | 27,80399 | 1,34E-07 | 1,38E-05 | down |
| ISM2 | -3,34234 | 2,335815 | 17,802 | 2,45E-05 | 0,001065 | down |
| F7 | -3,34669 | 1,514918 | 25,82347 | 3,74E-07 | 3,31E-05 | down |
| LPO | -3,38413 | 0,658845 | 17,4786 | 2,91E-05 | 0,001222 | down |
| SPACA3 | -3,73285 | 0,69541 | 26,09558 | 3,25E-07 | 2,99E-05 | down |
| COL2A1 | -4,07026 | 2,952042 | 15,13567 | 0,0001 | 0,003255 | down |
| IGF2 | -4,09139 | 10,26723 | 16,34069 | 5,29E-05 | 0,002 | down |
| SHISA9 | -4,23025 | 2,179578 | 13,56259 | 0,000231 | 0,006196 | down |
| CTCFL | 6,553869 | -0,46072 | 107,9965 | 2,69E-25 | 4,53E-22 | up |
| MSMB | 6,538138 | -1,28853 | 146,97 | 7,97E-34 | 6,70E-30 | up |
| PRSS2 | 5,521954 | 5,378901 | 95,66931 | 1,36E-22 | 1,90E-19 | up |
| FGL1 | 5,028575 | -0,56433 | 18,51601 | 1,68E-05 | 0,00077 | up |
| DHRS2 | 4,287907 | 3,518535 | 134,1777 | 5,00E-31 | 2,10E-27 | up |
| INSM1 | 4,102797 | 0,541862 | 115,2411 | 6,97E-27 | 1,67E-23 | up |
| SFTPA1 | 4,06775 | -2,44588 | 32,93039 | 9,55E-09 | 1,50E-06 | up |
| DLGAP1-AS5 | 4,048247 | -0,57028 | 40,26976 | 2,21E-10 | 5,17E-08 | up |
| NMUR2 | 3,938373 | 1,15499 | 19,33705 | 1,10E-05 | 0,00054 | up |
| ANXA10 | 3,874053 | -0,23797 | 20,11082 | 7,31E-06 | 0,000394 | up |
| TRPV6 | 3,872468 | -1,32451 | 76,12902 | 2,66E-18 | 1,94E-15 | up |
| ALOX15B | 3,800228 | 1,381661 | 144,1375 | 3,32E-33 | 1,86E-29 | up |
| TRHDE-AS1 | 3,769984 | -1,29469 | 43,4267 | 4,40E-11 | 1,23E-08 | up |
| PROK2 | 3,706385 | -0,18766 | 79,57812 | 4,64E-19 | 4,33E-16 | up |
| ITLN2 | 3,53214 | 4,128213 | 16,97764 | 3,78E-05 | 0,001515 | up |
| IL23A | 3,500289 | 2,574701 | 187,1549 | 1,33E-42 | 2,23E-38 | up |
| MUC6 | 3,491445 | 1,551816 | 46,20219 | 1,07E-11 | 3,32E-09 | up |
| HCAR2 | 3,412903 | 1,218427 | 89,01256 | 3,92E-21 | 4,40E-18 | up |
| SPINK4 | 3,371755 | 7,019219 | 46,05866 | 1,15E-11 | 3,45E-09 | up |
| TPH1 | 3,35571 | 1,62823 | 78,09765 | 9,81E-19 | 7,85E-16 | up |
| OSM | 3,343885 | 3,051896 | 118,4035 | 1,41E-27 | 3,97E-24 | up |
| EPHB6 | 3,234191 | 2,075846 | 107,3532 | 3,73E-25 | 5,70E-22 | up |
| HCAR3 | 3,227176 | -0,18835 | 50,60728 | 1,13E-12 | 4,04E-10 | up |
| FPR2 | 3,21101 | 1,179944 | 78,32849 | 8,73E-19 | 7,34E-16 | up |
| GALNTL6 | 3,167981 | 0,499166 | 39,10617 | 4,01E-10 | 9,12E-08 | up |
| FCAR | 3,105061 | -0,46862 | 66,04178 | 4,41E-16 | 2,75E-13 | up |
| IL1B | 3,104504 | 4,892791 | 133,5592 | 6,82E-31 | 2,29E-27 | up |
| TREM1 | 3,083244 | 2,425143 | 113,1264 | 2,02E-26 | 4,26E-23 | up |
| CCL3L3 | 3,073197 | 1,586639 | 93,99379 | 3,17E-22 | 4,09E-19 | up |
| SERPINB2 | 3,069586 | -0,56315 | 27,16668 | 1,87E-07 | 1,86E-05 | up |
| CXCL8 | 3,0684 | 7,159066 | 82,58646 | 1,01E-19 | 1,06E-16 | up |
| ALDH1A2 | 3,067712 | 2,569945 | 46,16757 | 1,09E-11 | 3,32E-09 | up |
| LRRC26 | 3,037124 | 3,86818 | 35,82274 | 2,16E-09 | 4,13E-07 | up |
| MMP8 | 3,012794 | -1,03582 | 42,21463 | 8,18E-11 | 2,22E-08 | up |
| KIF19 | 2,988411 | 1,80757 | 54,1078 | 1,90E-13 | 8,23E-11 | up |
| GALNT8 | 2,972395 | 3,931701 | 66,07011 | 4,35E-16 | 2,75E-13 | up |
| SFTPB | 2,967727 | -0,55447 | 17,63623 | 2,67E-05 | 0,001145 | up |
| KLK2 | 2,966366 | -1,48722 | 23,70913 | 1,12E-06 | 8,19E-05 | up |
| B3GNT6 | 2,896679 | 4,005632 | 28,39355 | 9,90E-08 | 1,06E-05 | up |
| KLK12 | 2,866115 | 2,123198 | 41,65165 | 1,09E-10 | 2,82E-08 | up |
| KLK3 | 2,744157 | 0,926972 | 30,02862 | 4,26E-08 | 4,94E-06 | up |
| MCEMP1 | 2,657231 | 0,71198 | 44,62927 | 2,38E-11 | 7,03E-09 | up |
| TCN1 | 2,622637 | 4,603879 | 23,56608 | 1,21E-06 | 8,68E-05 | up |
| HSPA2 | 2,621958 | 4,849714 | 109,7437 | 1,12E-25 | 2,08E-22 | up |
| LINC00261 | 2,602297 | 5,617267 | 53,99963 | 2,01E-13 | 8,43E-11 | up |
| NTSR1 | 2,577199 | 1,514506 | 28,15103 | 1,12E-07 | 1,19E-05 | up |
| CSF3 | 2,575744 | 1,896165 | 49,22808 | 2,28E-12 | 7,82E-10 | up |
| MYO18B | 2,57079 | -0,73007 | 22,85887 | 1,74E-06 | 0,000117 | up |
| BMPR1B | 2,538781 | 0,800926 | 24,98783 | 5,77E-07 | 4,68E-05 | up |
| BCL2A1 | 2,534538 | 2,508017 | 89,16 | 3,64E-21 | 4,37E-18 | up |
| NEFL | 2,520145 | 0,821707 | 17,3415 | 3,12E-05 | 0,001291 | up |
| AQP3 | 2,511201 | 5,200063 | 52,45801 | 4,40E-13 | 1,76E-10 | up |
| CABP7 | 2,500161 | -1,10651 | 76,67645 | 2,01E-18 | 1,54E-15 | up |
| CLC | 2,497639 | 1,834202 | 16,80254 | 4,15E-05 | 0,001615 | up |
| IDO1 | 2,486819 | 4,584091 | 42,00431 | 9,11E-11 | 2,43E-08 | up |
| CD300E | 2,485171 | 0,980048 | 53,94559 | 2,06E-13 | 8,46E-11 | up |
| MUC2 | 2,4848 | 10,13299 | 25,72518 | 3,94E-07 | 3,41E-05 | up |
| IGFALS | 2,4531 | 1,294438 | 30,72466 | 2,97E-08 | 3,73E-06 | up |
| PCSK1 | 2,449986 | 5,006325 | 25,54972 | 4,31E-07 | 3,66E-05 | up |
| GAPLINC | 2,440702 | -0,05462 | 79,97106 | 3,80E-19 | 3,76E-16 | up |
| SPP1 | 2,433067 | 7,4443 | 35,57023 | 2,46E-09 | 4,62E-07 | up |
| LINC00624 | 2,422453 | -0,66508 | 65,23022 | 6,66E-16 | 3,86E-13 | up |
| SLITRK6 | 2,41259 | 3,674565 | 21,68421 | 3,21E-06 | 0,0002 | up |
| FAM177B | 2,408214 | 1,381382 | 27,66456 | 1,44E-07 | 1,46E-05 | up |
| C5orf46 | 2,403265 | -0,82621 | 25,64291 | 4,11E-07 | 3,53E-05 | up |
| SLC18A1 | 2,399306 | 1,34902 | 30,35497 | 3,60E-08 | 4,41E-06 | up |
| UGT2B17 | 2,398501 | 5,199362 | 17,88775 | 2,34E-05 | 0,001024 | up |
| CALML3 | 2,394264 | -0,05969 | 33,89465 | 5,82E-09 | 9,50E-07 | up |
| CXCR1 | 2,382933 | 0,902113 | 34,15892 | 5,08E-09 | 8,46E-07 | up |
| FCGBP | 2,381963 | 9,583289 | 25,43724 | 4,57E-07 | 3,86E-05 | up |
| FCGR3B | 2,375037 | 1,934102 | 34,64831 | 3,95E-09 | 6,78E-07 | up |
| FFAR2 | 2,373922 | 1,795257 | 35,16208 | 3,03E-09 | 5,37E-07 | up |
| GRIP2 | 2,360868 | 1,631639 | 57,10061 | 4,14E-14 | 2,05E-11 | up |
| SERPINA1 | 2,359358 | 8,396542 | 59,02114 | 1,56E-14 | 8,20E-12 | up |
| REG4 | 2,350768 | 8,599081 | 18,12671 | 2,07E-05 | 0,000917 | up |
| AQP9 | 2,350032 | 2,411606 | 50,99988 | 9,24E-13 | 3,38E-10 | up |
| DGKB | 2,341544 | 0,150733 | 14,61816 | 0,000132 | 0,004026 | up |
| FER1L6 | 2,331399 | 2,585295 | 22,34659 | 2,28E-06 | 0,000151 | up |
| IL1RN | 2,330481 | 4,361533 | 79,40431 | 5,06E-19 | 4,48E-16 | up |
| SPDEF | 2,328064 | 4,719361 | 38,51829 | 5,42E-10 | 1,17E-07 | up |
| CLEC5A | 2,325823 | 1,602442 | 61,71811 | 3,96E-15 | 2,15E-12 | up |
| FPR1 | 2,316474 | 3,107271 | 52,23027 | 4,94E-13 | 1,93E-10 | up |
| IL6 | 2,310784 | 2,839235 | 34,56114 | 4,13E-09 | 7,02E-07 | up |
| CCL3 | 2,303979 | 2,528575 | 66,40707 | 3,67E-16 | 2,47E-13 | up |
| PADI4 | 2,299589 | -1,60767 | 37,0293 | 1,16E-09 | 2,39E-07 | up |
| BRINP3 | 2,296752 | 0,018168 | 15,90048 | 6,68E-05 | 0,002389 | up |
| REP15 | 2,287178 | 1,01102 | 25,65314 | 4,09E-07 | 3,52E-05 | up |
| UGT2B15 | 2,281053 | 2,351925 | 27,00576 | 2,03E-07 | 1,97E-05 | up |
| MUC5AC | 2,280261 | 5,156243 | 18,43751 | 1,76E-05 | 0,000794 | up |
| PCP4L1 | 2,263638 | -1,09111 | 23,09401 | 1,54E-06 | 0,000106 | up |
| PRKCG | 2,255081 | 1,507485 | 22,10605 | 2,58E-06 | 0,000166 | up |
| KLHL32 | 2,235109 | 1,710348 | 46,97671 | 7,18E-12 | 2,28E-09 | up |
| TNFRSF11B | 2,214173 | 4,655224 | 54,09733 | 1,91E-13 | 8,23E-11 | up |
| CD69 | 2,208421 | 2,41911 | 54,88052 | 1,28E-13 | 5,82E-11 | up |
| GRIN1 | 2,201408 | 1,372251 | 38,9834 | 4,27E-10 | 9,59E-08 | up |
| ADGRE3 | 2,171433 | -0,26503 | 31,67919 | 1,82E-08 | 2,53E-06 | up |
| CCL7 | 2,167743 | -1,72343 | 28,46191 | 9,56E-08 | 1,03E-05 | up |
| LINC00923 | 2,156195 | -0,61621 | 25,22713 | 5,10E-07 | 4,26E-05 | up |
| KIT | 2,154968 | 3,432019 | 67,3857 | 2,23E-16 | 1,56E-13 | up |
| KLK15 | 2,149118 | 0,778723 | 28,85726 | 7,79E-08 | 8,68E-06 | up |
| SMOC2 | 2,144154 | 6,575801 | 43,81559 | 3,61E-11 | 1,03E-08 | up |
| RHBDL3 | 2,140024 | 0,185422 | 24,08466 | 9,22E-07 | 6,92E-05 | up |
| IL24 | 2,132303 | 2,552578 | 30,25629 | 3,79E-08 | 4,52E-06 | up |
| GP2 | 2,132216 | 0,828269 | 12,52877 | 0,000401 | 0,009348 | up |
| EOMES | 2,121649 | -0,21028 | 38,94391 | 4,36E-10 | 9,65E-08 | up |
| WFDC2 | 2,119013 | 6,120442 | 32,19595 | 1,39E-08 | 1,99E-06 | up |
| FOLR1 | 2,116317 | 3,297186 | 18,0884 | 2,11E-05 | 0,000933 | up |
| CA9 | 2,116014 | 5,361479 | 24,56329 | 7,19E-07 | 5,57E-05 | up |
| S100A8 | 2,115313 | 2,67902 | 40,61381 | 1,85E-10 | 4,46E-08 | up |
| LINC01550 | 2,111539 | -0,82144 | 21,5884 | 3,38E-06 | 0,000208 | up |
| IL1A | 2,101449 | 1,832428 | 35,43082 | 2,64E-09 | 4,88E-07 | up |
| ADGRG3 | 2,09765 | 1,00088 | 52,01859 | 5,50E-13 | 2,10E-10 | up |
| CA8 | 2,094995 | 3,38174 | 27,84798 | 1,31E-07 | 1,35E-05 | up |
| IRX3 | 2,090712 | 0,014183 | 20,64524 | 5,53E-06 | 0,000317 | up |
| KLK11 | 2,08331 | 2,991322 | 26,00314 | 3,41E-07 | 3,10E-05 | up |
| TUBB4A | 2,080432 | -1,33873 | 35,55916 | 2,47E-09 | 4,62E-07 | up |
| SLC11A1 | 2,054186 | 3,134028 | 62,9798 | 2,09E-15 | 1,17E-12 | up |
| SLAIN1 | 2,04872 | 1,638257 | 30,54638 | 3,26E-08 | 4,06E-06 | up |
| CHRDL2 | 2,045845 | 3,189037 | 25,76261 | 3,86E-07 | 3,36E-05 | up |
| SLAMF9 | 2,030327 | -1,09217 | 32,80857 | 1,02E-08 | 1,57E-06 | up |
| CSF3R | 2,026936 | 3,250255 | 48,91356 | 2,67E-12 | 9,00E-10 | up |
| FOXI1 | 2,0257 | -1,37972 | 13,26613 | 0,00027 | 0,006981 | up |
| CYTL1 | 2,025068 | -0,58867 | 33,93568 | 5,70E-09 | 9,39E-07 | up |
| TOX | 2,023118 | 3,369679 | 49,82934 | 1,68E-12 | 5,88E-10 | up |
| GABRP | 2,016912 | 2,584865 | 15,81951 | 6,97E-05 | 0,002452 | up |
| PLEK | 2,002076 | 4,133152 | 55,54693 | 9,13E-14 | 4,39E-11 | up |
| CXCR2 | 1,992616 | 1,372496 | 32,30131 | 1,32E-08 | 1,93E-06 | up |
| VSIG8 | 1,992191 | -0,85088 | 18,93466 | 1,35E-05 | 0,000639 | up |
| PI15 | 1,990023 | 2,163206 | 31,25166 | 2,27E-08 | 3,03E-06 | up |
| MARCO | 1,9877 | 1,805881 | 15,28317 | 9,25E-05 | 0,00307 | up |
| CACNA2D2 | 1,9848 | 2,844076 | 41,57149 | 1,14E-10 | 2,90E-08 | up |
| RHOXF1-AS1 | 1,97306 | -0,85885 | 24,53075 | 7,31E-07 | 5,64E-05 | up |
| DOCK8-AS1 | 1,953459 | -1,31639 | 28,10362 | 1,15E-07 | 1,20E-05 | up |
| RAB26 | 1,946894 | 2,192661 | 28,58031 | 8,99E-08 | 9,82E-06 | up |
| SYCP2L | 1,942376 | -1,45321 | 20,39233 | 6,31E-06 | 0,000355 | up |
| ATOH1 | 1,93972 | 4,335288 | 19,55935 | 9,75E-06 | 0,000499 | up |
| WNT7B | 1,939259 | 0,600655 | 18,97321 | 1,33E-05 | 0,00063 | up |
| NPW | 1,936406 | 1,768535 | 20,6379 | 5,55E-06 | 0,000317 | up |
| MEFV | 1,934539 | -0,20158 | 35,32965 | 2,78E-09 | 5,03E-07 | up |
| GFI1 | 1,920092 | 1,779787 | 48,81647 | 2,81E-12 | 9,27E-10 | up |
| ABCC8 | 1,918651 | -1,94305 | 25,5516 | 4,31E-07 | 3,66E-05 | up |
| ABCA4 | 1,916044 | -0,49319 | 18,93763 | 1,35E-05 | 0,000639 | up |
| TDO2 | 1,915631 | 3,63995 | 19,48277 | 1,02E-05 | 0,000513 | up |
| SHISA3 | 1,914187 | 0,648883 | 30,91042 | 2,70E-08 | 3,52E-06 | up |
| FCN1 | 1,91252 | 1,441546 | 35,18684 | 3,00E-09 | 5,36E-07 | up |
| PAEP | 1,901564 | -1,52238 | 16,95671 | 3,82E-05 | 0,001524 | up |
| LINC02345 | 1,894643 | -1,31903 | 23,9391 | 9,94E-07 | 7,40E-05 | up |
| SYT5 | 1,890027 | -1,61209 | 36,85433 | 1,27E-09 | 2,58E-07 | up |
| TRIM7 | 1,88324 | 2,205449 | 23,46645 | 1,27E-06 | 9,06E-05 | up |
| MCHR1 | 1,872195 | -1,9224 | 26,44641 | 2,71E-07 | 2,52E-05 | up |
| SDK2 | 1,869381 | 1,698285 | 30,29781 | 3,71E-08 | 4,48E-06 | up |
| G0S2 | 1,866385 | 4,08902 | 44,28531 | 2,84E-11 | 8,23E-09 | up |
| SHOX2 | 1,8638 | -1,0524 | 20,65103 | 5,51E-06 | 0,000317 | up |
| APOBEC3A | 1,863161 | -0,49826 | 32,97049 | 9,36E-09 | 1,48E-06 | up |
| ANGPTL4 | 1,85686 | 3,004717 | 51,3977 | 7,54E-13 | 2,82E-10 | up |
| RASD1 | 1,854215 | 3,997863 | 30,06265 | 4,18E-08 | 4,92E-06 | up |
| COLCA1 | 1,849264 | 2,97342 | 18,57022 | 1,64E-05 | 0,000755 | up |
| VMO1 | 1,849188 | 0,900268 | 65,47614 | 5,88E-16 | 3,53E-13 | up |
| PRND | 1,843216 | -0,44039 | 19,54586 | 9,82E-06 | 0,000501 | up |
| CNDP1 | 1,83684 | -1,51195 | 14,26073 | 0,000159 | 0,004663 | up |
| TMEM61 | 1,832405 | 0,578223 | 29,87504 | 4,61E-08 | 5,31E-06 | up |
| PDE4B | 1,831428 | 4,201503 | 55,13217 | 1,13E-13 | 5,26E-11 | up |
| SLITRK5 | 1,829647 | -1,29401 | 14,66481 | 0,000128 | 0,003941 | up |
| CXCL6 | 1,828938 | 2,164892 | 26,98316 | 2,05E-07 | 1,97E-05 | up |
| DBH | 1,828623 | -1,71418 | 23,38484 | 1,33E-06 | 9,33E-05 | up |
| OPRD1 | 1,828331 | 0,379514 | 17,96383 | 2,25E-05 | 0,000994 | up |
| PTPRN | 1,821654 | 0,773135 | 33,26609 | 8,04E-09 | 1,29E-06 | up |
| PPP4R4 | 1,820075 | -0,72224 | 15,89691 | 6,69E-05 | 0,002389 | up |
| VEPH1 | 1,811028 | -0,42201 | 30,34194 | 3,62E-08 | 4,41E-06 | up |
| ERMN | 1,808293 | -0,54483 | 31,64048 | 1,86E-08 | 2,56E-06 | up |
| RAMP1 | 1,802731 | 3,761521 | 26,47676 | 2,67E-07 | 2,49E-05 | up |
| IL11 | 1,788986 | 2,79936 | 19,68839 | 9,12E-06 | 0,00047 | up |
| APCDD1L | 1,784901 | -0,30936 | 15,15814 | 9,89E-05 | 0,003223 | up |
| LINC01301 | 1,783648 | 1,097853 | 19,74484 | 8,85E-06 | 0,000461 | up |
| TNFAIP6 | 1,774957 | 2,489933 | 29,74307 | 4,93E-08 | 5,64E-06 | up |
| TPSG1 | 1,77424 | 2,941657 | 17,80904 | 2,44E-05 | 0,001064 | up |
| C2CD4C | 1,765959 | -0,24507 | 42,76328 | 6,18E-11 | 1,70E-08 | up |
| TFF1 | 1,754809 | 6,651517 | 20,43576 | 6,17E-06 | 0,000348 | up |
| GPAT2 | 1,748345 | 1,044128 | 21,77054 | 3,07E-06 | 0,000192 | up |
| COL16A1 | 1,738778 | 6,028392 | 37,5107 | 9,09E-10 | 1,94E-07 | up |
| RAB3B | 1,731621 | 2,051889 | 30,03521 | 4,24E-08 | 4,94E-06 | up |
| C4BPB | 1,727645 | 3,22208 | 37,28562 | 1,02E-09 | 2,15E-07 | up |
| S100A12 | 1,726571 | -1,07737 | 12,92193 | 0,000325 | 0,007985 | up |
| ELAPOR1 | 1,72132 | 7,129136 | 28,61457 | 8,83E-08 | 9,71E-06 | up |
| CCDC187 | 1,712637 | 0,25025 | 16,09313 | 6,03E-05 | 0,00221 | up |
| MMP1 | 1,706136 | 7,026455 | 16,93141 | 3,88E-05 | 0,001534 | up |
| DNER | 1,703341 | -0,76426 | 19,13795 | 1,22E-05 | 0,000581 | up |
| CBFA2T3 | 1,697924 | 2,83811 | 25,86541 | 3,66E-07 | 3,27E-05 | up |
| PTGS2 | 1,696983 | 4,159418 | 24,13521 | 8,98E-07 | 6,77E-05 | up |
| CITED1 | 1,692605 | 0,5377 | 19,91371 | 8,10E-06 | 0,000431 | up |
| LUCAT1 | 1,680162 | -1,16408 | 28,13914 | 1,13E-07 | 1,19E-05 | up |
| TMEM132B | 1,677575 | -1,08243 | 30,2371 | 3,82E-08 | 4,53E-06 | up |
| FAM174B | 1,662487 | 4,246364 | 58,39299 | 2,15E-14 | 1,09E-11 | up |
| CSF2 | 1,6602 | 0,205266 | 12,85507 | 0,000337 | 0,008192 | up |
| MYO3B | 1,653667 | -0,23136 | 20,97904 | 4,64E-06 | 0,000275 | up |
| GPIHBP1 | 1,636906 | -0,7281 | 20,14105 | 7,19E-06 | 0,000389 | up |
| CBLIF | 1,626008 | 0,899715 | 12,39178 | 0,000431 | 0,009841 | up |
| MTUS2 | 1,625591 | -1,46959 | 29,18396 | 6,58E-08 | 7,43E-06 | up |
| SHF | 1,600603 | 3,578071 | 35,87569 | 2,10E-09 | 4,11E-07 | up |
| S100A9 | 1,593596 | 5,700923 | 23,56454 | 1,21E-06 | 8,68E-05 | up |
| LILRA2 | 1,59267 | 0,058138 | 27,97245 | 1,23E-07 | 1,28E-05 | up |
| IL1R2 | 1,592601 | 3,545289 | 22,28372 | 2,35E-06 | 0,000154 | up |
| KANK4 | 1,592484 | 0,512178 | 19,21107 | 1,17E-05 | 0,000564 | up |
| CCL4 | 1,591374 | 2,660317 | 35,36474 | 2,73E-09 | 5,00E-07 | up |
| FZD9 | 1,590446 | 0,216994 | 13,97593 | 0,000185 | 0,005254 | up |
| RIMS3 | 1,587564 | 2,444207 | 38,64092 | 5,09E-10 | 1,11E-07 | up |
| VNN2 | 1,587332 | 1,345038 | 21,33225 | 3,86E-06 | 0,000235 | up |
| DRP2 | 1,586733 | 1,129174 | 18,37597 | 1,81E-05 | 0,000813 | up |
| KCNJ15 | 1,575703 | 1,077863 | 17,7271 | 2,55E-05 | 0,0011 | up |
| PTGER2 | 1,5689 | 2,604613 | 33,37426 | 7,60E-09 | 1,23E-06 | up |
| CAPN9 | 1,567167 | 3,173422 | 17,49419 | 2,88E-05 | 0,001215 | up |
| ADRB1 | 1,559358 | -0,06301 | 13,80894 | 0,000202 | 0,005598 | up |
| CHST6 | 1,554401 | 1,572121 | 21,54347 | 3,46E-06 | 0,000212 | up |
| NRG4 | 1,551987 | -1,1974 | 22,61535 | 1,98E-06 | 0,000132 | up |
| SH2D6 | 1,55197 | -0,15541 | 13,0188 | 0,000308 | 0,007695 | up |
| KIR2DL4 | 1,551122 | -1,55339 | 13,36109 | 0,000257 | 0,00672 | up |
| DUSP4 | 1,547925 | 4,950633 | 21,77182 | 3,07E-06 | 0,000192 | up |
| WNT9A | 1,538578 | 1,241943 | 34,41309 | 4,46E-09 | 7,50E-07 | up |
| ARL9 | 1,536931 | -1,14751 | 22,69541 | 1,90E-06 | 0,000127 | up |
| SERPINE1 | 1,534047 | 5,576392 | 20,93044 | 4,76E-06 | 0,000281 | up |
| SPTB | 1,531814 | 0,171744 | 32,26661 | 1,34E-08 | 1,95E-06 | up |
| LINC00239 | 1,529825 | 1,304335 | 32,42219 | 1,24E-08 | 1,83E-06 | up |
| CLEC4A | 1,528494 | 1,766548 | 41,40754 | 1,24E-10 | 3,10E-08 | up |
| RARRES1 | 1,525888 | 4,772301 | 23,60829 | 1,18E-06 | 8,56E-05 | up |
| GPR84 | 1,520797 | 0,683975 | 27,43404 | 1,63E-07 | 1,63E-05 | up |
| CCL4L2 | 1,519394 | 1,825018 | 22,9996 | 1,62E-06 | 0,00011 | up |
| HYAL1 | 1,517747 | 3,19684 | 16,32051 | 5,35E-05 | 0,002017 | up |
| GFPT2 | 1,517124 | 2,832379 | 20,89703 | 4,85E-06 | 0,000285 | up |
| FAM189A2 | 1,517085 | 0,027511 | 21,94312 | 2,81E-06 | 0,00018 | up |
| CASS4 | 1,515865 | 0,850857 | 35,97659 | 2,00E-09 | 3,95E-07 | up |
| SRGN | 1,513779 | 5,696485 | 31,95392 | 1,58E-08 | 2,23E-06 | up |
